# Psychological correlates of problematic pornography use: A systematic review and meta-analysis of measurement and conceptualization differences

**DOI:** 10.1016/j.abrep.2026.100741

**Published:** 2026-08-26

**Authors:** Sonay Sheikhi, Shahram Mohammadkhani, Mehdi Akbari

**Affiliations:** Department of Clinical Psychology, Faculty of Psychology and Education, Kharazmi University, Tehran, Iran

**Keywords:** Problematic pornography use, Moral incongruence, Behavioral dysregulation, Compulsive sexual behavior, Meta-analysis, Measurement heterogeneity

## Abstract

Problematic Pornography Use (PPU) has been linked to a wide range of psychological correlates, however findings across studies remain inconsistent. One possible explanation is that PPU has been operationalized using conceptually distinct approaches emphasizing either behavioral dysregulation or subjective distress, including moral incongruence and negative self-evaluation. This systematic review and meta-analysis synthesized psychological correlates of PPU and examined whether associations varied across different operationalizations of the construct.A systematic search identified 97 articles comprising 111 independent studies/samples. Random-effects meta-analyses were conducted, and studies were coded according to the dominant measurement orientation of PPU instruments, distinguishing dysregulation-oriented from distress-oriented operationalizations. Exploratory moderator and subgroup analyses examined whether differences in PPU operationalization and measurement orientation contributed to effect-size variability. Prediction intervals were calculated to characterize the expected dispersion of effects across future comparable studies.PPU was significantly associated with craving, emotion dysregulation, depression, loneliness, sexual shame, and moral disapproval. Substantial heterogeneity was observed, and prediction intervals were often wide. Dysregulation-oriented measures tended to show relatively stronger associations with craving and impulsivity, whereas distress-oriented measures tended to show relatively stronger associations with shame, religiosity, and moral conflict.Exploratory analyses suggested that measurement differences may partly contribute to variability in observed correlates.Psychological correlates of PPU appear to vary partly according to how the construct is conceptualized and measured. Distinguishing distress-oriented from dysregulation-oriented operationalizations may help clarify inconsistencies and improve theoretical precision in future research.

## Highlights

• PPU correlates varied across operational definitions.
• Distress- and dysregulation-oriented measures showed distinct patterns.
• Craving and impulsivity were strongest in dysregulation measures.
• Shame and moral conflict were more pronounced in distress measures.
• High heterogeneity limited universal interpretation of pooled effects.

## 1 Introduction

### 1.1 Pornography use in the digital context

The rapid expansion of digital technologies, mobile internet access, and algorithmically curated online platforms has substantially transformed the accessibility, frequency, and patterns of pornography consumption. Online sexual content is now widely accessible through mobile technologies, high-speed internet, and algorithmically curated digital platforms, contributing to its integration into everyday digital life (Kohut et al., 2020). Large-scale epidemiological and cross-cultural studies indicate that pornography use is prevalent across genders and age groups, particularly among adolescents and young adults, and that for most individuals such use is not associated with clinically significant impairment or psychopathology (Bőthe et al., 2020; Kohut et al., 2020). Accordingly, pornography use per se should not be equated with psychopathology or behavioral addiction.

At the same time, a subset of individuals report persistent difficulties related to their pornography use, including perceived loss of control, excessive preoccupation, subjective distress, compulsive engagement, and interference with daily functioning (Bőthe, Tóth-Király, Bella, et al., 2021). These experiences are commonly described under the umbrella term problematic pornography use (PPU). Importantly, PPU does not constitute a formal psychiatric diagnosis but rather refers to a heterogeneous set of self-reported difficulties that may arise from multiple and potentially overlapping psychological processes (Bőthe, Tóth-Király, Griffiths, et al., 2021; Grubbs & Perry, 2019).

### 1.2 Conceptual and measurement heterogeneity in PPU

One of the central challenges in the PPU literature concerns substantial heterogeneity in how the construct is conceptualized and operationalized (Bőthe, Tóth-Király, Bella, et al., 2021; Grubbs & Perry, 2019; Kohut et al., 2020). Widely used instruments differ markedly in their conceptual emphasis and symptom content. Some measures primarily assess behavioral dysregulation, including impaired control, compulsive engagement, salience, craving, and functional impairment (e.g., Problematic Pornography Consumption Scale [PPCS]; Short Internet Addiction Test adapted for pornography use [s-IAT-sex]; Bőthe et al., 2020; Wéry et al., 2016). In contrast, other instruments emphasize subjective distress and evaluative responses, including guilt, shame, self-perceived addiction, and moral incongruence (e.g., Cyber Pornography Use Inventory [CPUI]; Grubbs et al., 2010; Grubbs et al., 2018).

These differences raise important concerns for both primary studies and quantitative syntheses. Studies labeled as assessing “problematic pornography use” may, in practice, capture partially distinct aspects of experience, thereby contributing to variability in observed associations with psychological variables. For example, associations with religiosity, shame, or moral incongruence may be more pronounced in studies using distress-oriented instruments, whereas associations with impulsivity, craving, and emotion dysregulation may be stronger in studies emphasizing behavioral dysregulation and impaired control (Bőthe et al., 2020; Perry & Whitehead, 2019). Consequently, pooled meta-analytic estimates may obscure important conceptual differences if measurement variability is not explicitly considered.

Such variability also raises concerns regarding the generalizability of pooled effect estimates across studies, as substantial between-study heterogeneity may reflect differences in conceptualization and measurement rather than random variation alone. Despite these concerns, relatively few quantitative syntheses have systematically examined the extent to which operationalization differences contribute to variability in reported correlates of PPU.

### 1.3 Theoretical frameworks and their scope

Several theoretical models have been proposed to account for problematic pornography use, each emphasizing somewhat different psychological processes. The Interaction of Person–Affect–Cognition–Execution (I-PACE) model conceptualizes problematic online behaviors as emerging from dynamic interactions among individual predispositions, affective responses, cognitive biases, reinforcement learning processes, and executive functioning deficits (Brand et al., 2016, 2019). Although the I-PACE model was developed as a general framework for addictive online behaviors rather than specifically for PPU, it has frequently been applied to explain dysregulation-related aspects of problematic pornography use, including cue-reactivity, craving, impaired inhibitory control, and compulsive engagement (Antons & Brand, 2018; Brand et al., 2019).

The Pornography Problems due to Moral Incongruence (PPMI) model, by comparison, emphasizes the role of value-based conflict and subjective distress, proposing that individuals may perceive their pornography use as problematic due to discrepancies between their behavior and internalized moral or religious standards (Grubbs et al., 2018). Within this perspective, self-perceived problematic use may arise even in the absence of pronounced behavioral dysregulation. Recent conceptual work has increasingly emphasized the importance of distinguishing behavioral dysregulation from subjective moral distress when interpreting self-reported problematic pornography use (Bőthe et al., 2020; Grubbs & Perry, 2019; Kohut et al., 2020).

Importantly, these theoretical perspectives are not necessarily mutually exclusive. Rather, they appear to emphasize partially overlapping but conceptually distinguishable processes that may contribute to self-reported PPU in different individuals and contexts (Bőthe et al., 2020; Kohut et al., 2020). Recent conceptual discussions suggest that problematic pornography use is best understood as a multidimensional construct encompassing behavioral dysregulation, affect-regulation difficulties, and moral-distress-related experiences rather than a single homogeneous syndrome (Akbari et al., 2026; Bőthe et al., 2020; Grubbs & Perry, 2019; Kohut et al., 2020).

At the same time, both the I-PACE and PPMI models are inherently process-oriented and imply temporal and developmental dynamics that cannot be directly tested using cross-sectional correlational evidence. Accordingly, the present study does not aim to evaluate or adjudicate between these theoretical models. Instead, it adopts a measurement-informed perspective, using these frameworks primarily as conceptual reference points for interpreting patterns of associations observed in the literature.

A growing body of research has identified a wide range of psychological correlates associated with PPU, including impulsivity, emotion dysregulation, craving, loneliness, depression, anxiety, interpersonal difficulties, shame, and moral incongruence (Antons & Brand, 2018; Bőthe, Tóth-Király, Griffiths, et al., 2021; Grubbs & Perry, 2019). Accumulating evidence further suggests that negative affect, emotional distress, and emotion-regulation difficulties are consistently associated with higher levels of problematic pornography use, although the directionality of these associations remains insufficiently established because most available evidence is cross-sectional (Antons & Brand, 2018; Bőthe, Tóth-Király, Bella, et al., 2021; Brand et al., 2019). Comparable patterns have also been reported across diverse populations and cultural settings, suggesting that several psychological correlates of PPU demonstrate considerable cross-cultural consistency (Akbari et al., 2024; Bőthe et al., 2020; Kohut et al., 2020).

One prominent line of research has focused on dysregulation-related correlates of PPU, particularly impulsivity, deficient inhibitory control, craving, and emotion regulation difficulties (Antons & Brand, 2018; Bőthe, Tóth-Király, Griffiths, et al., 2021). Within this perspective, PPU is conceptualized in terms of maladaptive reward processing and diminished self-regulatory capacity, broadly aligning with behavioral addiction frameworks (Brand et al., 2016, 2019; Robinson & Berridge, 2008). Repeated exposure to sexually explicit material has been proposed to increase cue-reactivity and craving, potentially contributing to compulsive patterns of engagement and impaired control over use.

In parallel, a growing body of literature has emphasized distress-related correlates of PPU, particularly shame, guilt, scrupulosity, and moral incongruence (Akbari et al., 2024; Grubbs et al., 2018; Perry & Whitehead, 2019). These constructs reflect negative self-evaluative and affective responses to pornography use that may be shaped by personal values, cultural norms, and religious beliefs. From this perspective, reports of “problematic” pornography use may arise not solely from compulsive dysregulation but from perceived discrepancies between one's behavior and internalized standards. Empirical evidence suggests that distress-related variables may be strongly associated with self-perceived PPU even when behavioral indicators of dysregulation are relatively weak (Bőthe et al., 2020; Grubbs et al., 2018).

Despite these advances, the literature remains conceptually and methodologically fragmented. In many studies, dysregulation-related and distress-related correlates are examined under a common PPU label without explicit differentiation between the underlying processes they may reflect. This has contributed to ongoing debate regarding whether observed associations primarily reflect compulsive engagement, subjective distress, or varying combinations of these processes (Bőthe, Tóth-Király, Bella, et al., 2021; Grubbs & Perry, 2019).

A key source of this ambiguity may lie in measurement heterogeneity. Widely used PPU instruments differ substantially in their conceptual emphasis, with some prioritizing behavioral dysregulation and others emphasizing subjective distress or moral conflict. Consequently, observed associations between PPU and psychological variables may partly reflect the specific content and conceptual orientation of the measures employed rather than uniform underlying relationships across studies (Bőthe, Tóth-Király, Griffiths, et al., 2021; Kohut et al., 2020).

Taken together, existing evidence suggests that PPU is associated with a heterogeneous constellation of psychological correlates spanning both dysregulation-related and distress-related domains. However, it remains unclear whether these associations generalize consistently across operationalizations or whether they are influenced, at least in part, by differences in conceptualization and measurement. Although previous reviews and meta-analyses have summarized the psychological correlates of pornography use and problematic pornography use, none has systematically evaluated whether these associations vary according to the conceptual orientation and measurement characteristics of the instruments used to assess PPU (Akbari et al., 2024; Bőthe et al., 2020; Kohut et al., 2020).

### 1.4 The present study

To the best of our knowledge, no previous meta-analysis has systematically examined whether associations between psychological correlates and problematic pornography use vary according to differences in conceptualization and measurement approach.In light of these considerations, the present study aimed to provide a comprehensive meta-analytic synthesis of psychological correlates associated with PPU while explicitly accounting for measurement heterogeneity.

Rather than assuming that PPU represents a single homogeneous construct or attempting to test predefined etiological pathways, the study adopts a measurement-informed perspective. Specifically, the objectives were to: (a) estimate pooled associations between PPU and major psychological correlates using two-level random-effects meta-analytic models; (b) quantify between-study heterogeneity and evaluate the robustness and generalizability of pooled effects using prediction intervals; and (c) examine whether the magnitude of these associations differed according to the conceptual emphasis of PPU measures (i.e., behavioral dysregulation-oriented versus distress-oriented operationalizations).

Importantly, all analyses were interpreted as correlational and descriptive in nature. Differences observed across operationalizations were treated as indicative of variability in measurement emphasis rather than as evidence for distinct etiological pathways. Accordingly, the findings are intended primarily to clarify patterns within the existing literature and to generate theoretically informed hypotheses for future longitudinal, experimental, and mechanistic research.

## 2 Method

### 2.1 Study design

This study was conducted as a systematic review and meta-analysis in accordance with the Preferred Reporting Items for Systematic Reviews and Meta-Analyses (PRISMA 2020) guidelines (Page et al., 2021). Before study selection, a review protocol was developed that specified the eligibility criteria, literature search strategy, data extraction procedures, coding framework, classification of problematic pornography use (PPU) instruments, and planned statistical analyses. Particular attention was given to construct validity and potential measurement heterogeneity across studies.

The methodological rigor of the review was informed by the AMSTAR 2 framework for systematic reviews (Shea et al., 2017), whereas the methodological quality of individual primary studies was evaluated separately using the quantitative assessment criteria developed by Kmet et al. (2004).

Given the structure of the extracted data, a conventional two-level random-effects meta-analytic model was considered the most appropriate analytical approach. Effect sizes were analyzed at the level of psychological constructs, with each study contributing a single independent effect size for each construct whenever possible. Because relatively few studies contributed multiple statistically dependent effect sizes within the same construct, the dependency structure required to justify multilevel (three-level) meta-analysis was not present (Van den Noortgate et al., 2013).

Accordingly, sampling variance was modeled at Level 1 and between-study heterogeneity at Level 2. Random-effects models were estimated using restricted maximum likelihood (REML), which provides efficient and relatively unbiased estimation of between-study variance under random-effects assumptions (Viechtbauer, 2010). Random-effects modeling was selected because true effect sizes were expected to vary across studies owing to differences in populations, sampling procedures, measurement instruments, cultural contexts, and study designs.

### 2.2 Coding of study variables

Information from all eligible studies was extracted using a standardized coding protocol developed before data extraction. Extracted variables included author(s), publication year, country, study design, sample size, mean age, gender distribution, timing relative to the COVID-19 pandemic (pre-, during-, or post-pandemic), PPU measurement instrument, psychological correlates assessed, reliability coefficients of study measures, and other relevant sample characteristics.

Timing relative to the COVID-19 pandemic was coded because pandemic-related social restrictions, changes in daily routines, and increased online activity may have influenced both pornography use patterns and associated psychological functioning.

Psychological correlates were subsequently grouped into broader conceptual domains (e.g., personality traits, emotional states, attachment, emotion regulation, impulsivity, craving, and sexuality-related constructs) to facilitate quantitative synthesis while preserving conceptual consistency across studies.

Coding was conducted independently by two reviewers using the predefined coding manual. Any discrepancies were resolved through discussion and consensus, with consultation from a third reviewer when necessary. Detailed characteristics of all included studies are presented in Table S1 (Supplementary Materials).

### 2.3 Literature search strategy

A comprehensive systematic literature search was conducted across multiple electronic databases, including PubMed/MEDLINE, Scopus, Web of Science, andsupplemented by searches of Google Scholar, regional databases (SID, Magiran, and Noormags), and grey literature sources, including the Open Access Theses and Dissertations (OATD) database.

The search covered studies published between January 2012 and December 2025 and included publications in English and Persian.

Search strategies combined controlled vocabulary (where available) and free-text keywords related to problematic pornography use (e.g., *problematic pornography use*, *pornography addiction*, *compulsive pornography use*, *problematic online pornography use*, *cyber pornography*) with terms referring to psychological correlates (e.g., *impulsivity*, *emotion regulation*, *attachment*, *craving*, *moral incongruence*, *depression*, *anxiety*, and *personality*). Boolean operators, truncation symbols, and database-specific search syntax were adapted as appropriate for each database. Complete search strategies for all databases are provided in the Supplementary Materials.

To minimize the risk of publication bias and maximize study retrieval, additional search procedures included backward reference searching, forward citation tracking, manual screening of reference lists from eligible articles and previous reviews, and searches of grey literature. Database search alerts were maintained throughout the review process until completion of the final search update.

A final literature search update was conducted between September and December 2025 to identify newly published studies. One additional eligible study meeting all inclusion criteria was identified during this update and incorporated into the final meta-analysis.

### 2.4 Eligibility criteria

Studies were considered eligible for inclusion if they met all of the following criteria: (a) reported original quantitative data; (b) examined the association between problematic pornography use (PPU), or conceptually related constructs (e.g., compulsive pornography use, compulsive sexual behavior involving pornography, hypersexual behavior involving pornography, or self-perceived pornography addiction), and at least one psychological correlate; (c) were published in English or Persian as peer-reviewed journal articles or eligible grey literature (e.g., doctoral dissertations or master's theses); (d) provided sufficient statistical information to calculate or convert effect sizes to Pearson's correlation coefficient (r); and (e) assessed PPU using multi-item instruments with previously reported psychometric evidence.

Studies were excluded if they consisted exclusively of qualitative data, case reports, reviews, editorials, conference abstracts lacking sufficient statistical information, or duplicate publications derived from the same dataset. When multiple reports were based on overlapping samples, the publication providing the most complete data or largest sample was retained.

A minimum sample size of 10 participants was established to maximize study inclusion while avoiding exclusion of early or relatively small investigations. Because very small samples may produce unstable effect size estimates, additional sensitivity analyses excluding studies with sample sizes below 50 participants were conducted to evaluate the robustness of the pooled estimates.

### 2.5 Study selection and data extraction

**Fig. 1:**
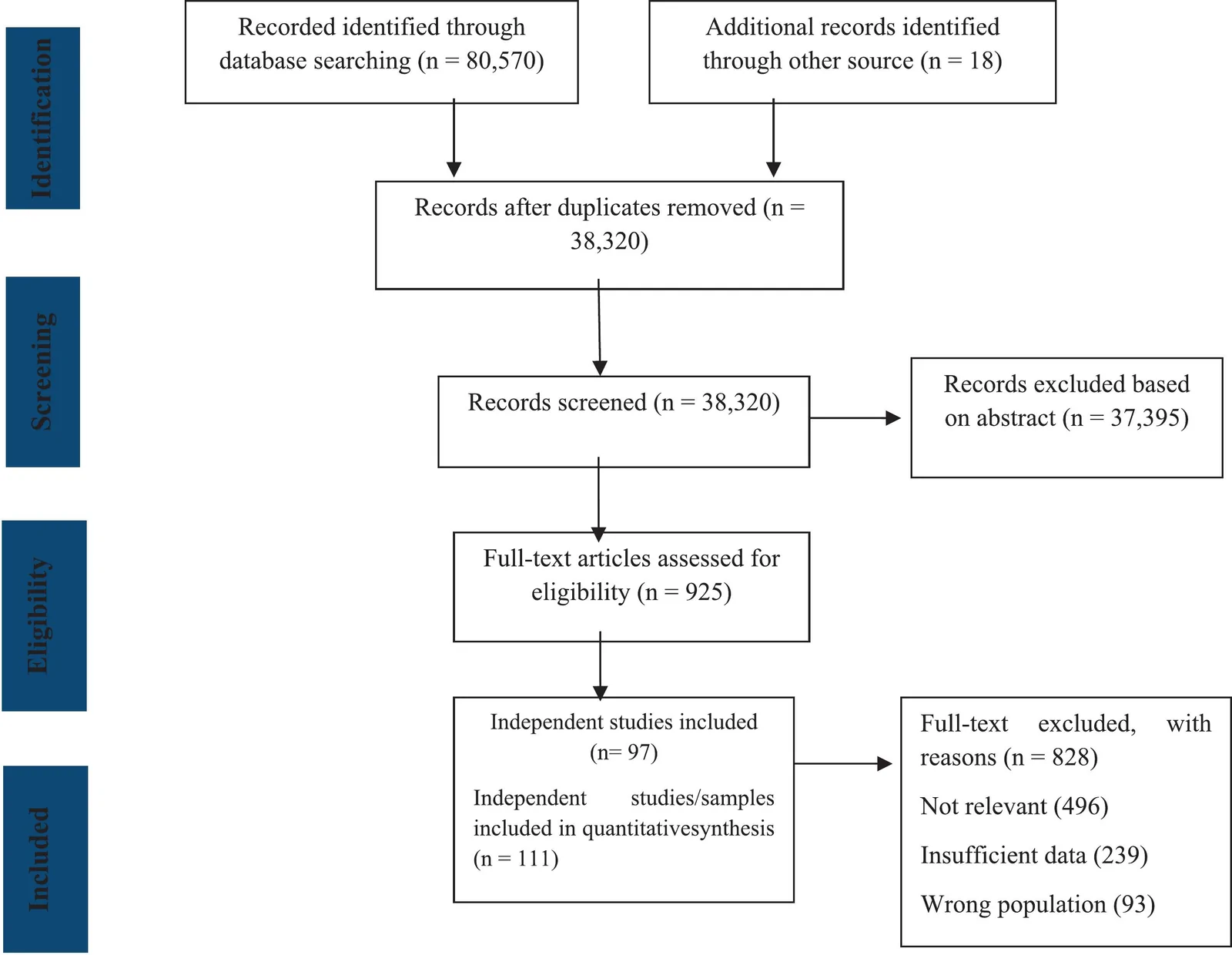
PRISMA flow diagram illustrating study identification, screening, eligibility, and inclusion.

Study selection was performed in accordance with the PRISMA 2020 recommendations (Page et al., 2021). The numbers of records identified, screened, excluded with reasons, and retained for review and quantitative synthesis are summarized in the PRISMA flow diagram (Fig. 1).

The number of studies and participants contributing to each specific meta-analytic model is reported separately in the corresponding results tables.Study selection involved two sequential screening stages. First, titles and abstracts retrieved from the database searches were independently screened by two reviewers to identify potentially eligible studies. Second, the full texts of all potentially relevant articles were independently evaluated against the predefined eligibility criteria. Disagreements at either stage were resolved through discussion and consensus, and consultation with a third reviewer was undertaken when consensus could not be reached.

Data extraction was conducted using a standardized data extraction form developed specifically for the present review. Extracted information included study characteristics, such as publication year, country, and study design; sample characteristics, such as sample size, mean age, and gender distribution; PPU measurement instruments; psychological correlates assessed; and reported associations between PPU and psychological variables.

To minimize statistical dependence among effect sizes, only one independent effect size was retained for each psychological construct within each study whenever possible. When multiple statistically equivalent effect sizes representing the same construct were reported, they were combined using established meta-analytic procedures or averaged after transformation to Fisher's z values when appropriate.

Age-related information was extracted whenever available. Specifically, mean age and corresponding standard deviations (SDs) were recorded for each study. Because age variability was inconsistently reported across the literature, no statistical imputation procedures were applied for missing SD values in order to avoid introducing additional assumptions into the analyses.

To summarize participant characteristics across studies, a sample-size-weighted mean age was calculated using all studies reporting mean age. Given the substantial proportion of studies that did not report age variability, a pooled standard deviation could not be estimated reliably. Consequently, only the weighted mean age is reported, and between-study variability in participant age should be interpreted cautiously.

### 2.6 Quality assessment

The methodological quality of the included studies was evaluated using the Standard Quality Assessment Criteria for Evaluating Primary Research Papers developed by Kmet et al. (2004). This instrument assesses multiple methodological domains, including clarity of study objectives, appropriateness of study design, sampling procedures, validity and reliability of measurement instruments, adequacy of statistical analyses, control of potential bias, and interpretation of findings.

Each study was independently evaluated by two reviewers using the predefined quality assessment criteria. Disagreements in quality ratings were resolved through discussion and consensus. Higher scores indicated greater methodological quality.

Quality assessment was conducted to describe the overall methodological characteristics of the evidence base and to inform sensitivity analyses. Quality scores were not used as inclusion or exclusion criteria, thereby minimizing the risk of selectively excluding studies on the basis of reporting quality alone.

Detailed quality assessment results for all included studies are presented in Table S2 (Supplementary Materials).

### 2.7 Effect size metric

Pearson's correlation coefficient (**r**) was selected as the common effect size metric for all analyses because the primary objective was to quantify the magnitude of associations between problematic pornography use (PPU) and psychological correlates across studies. When studies reported alternative effect size indices, such as standardized mean differences, odds ratios, or regression coefficients with sufficient accompanying statistical information, these statistics were converted to Pearson's r using established meta-analytic conversion procedures (Borenstein et al., 2009).

To stabilize sampling variances and improve the normality of the sampling distribution, all correlation coefficients were transformed to Fisher's z values prior to statistical analysis. Following meta-analysis, pooled estimates and corresponding confidence intervals were back-transformed to Pearson's *r* to facilitate interpretation.

The magnitude of pooled correlations was interpreted descriptively using the guidelines proposed by Evans (1996). These benchmarks were used solely to facilitate interpretation of effect sizes and were not treated as strict thresholds for substantive significance.

### 2.8 Classification of PPU measurement instruments

Because considerable variability exists in the conceptualization and operationalization of problematic pornography use (PPU), a priori classification of measurement instruments was undertaken to facilitate interpretation of measurement-related heterogeneity across studies.

Instruments were classified as behavioral dysregulation-oriented when their primary emphasis was on impaired control, compulsive engagement, craving, salience, persistence despite adverse consequences, or functional impairment associated with pornography use. Conversely, instruments were classified as distress-oriented when they primarily assessed subjective distress, guilt, shame, self-perceived addiction, moral incongruence, or other evaluative responses to pornography use.

Instrument classification was based on the original scale descriptions, published validation studies, and their dominant conceptual emphasis rather than individual item content alone. Because several instruments include elements reflecting both behavioral dysregulation and subjective distress, the resulting categories should be regarded as heuristic conceptual groupings rather than mutually exclusive or definitive classifications.

The primary purpose of this classification was not to distinguish etiological subtypes of PPU, but to evaluate whether differences in measurement emphasis contributed to variability in observed associations across studies.

### 2.9 Meta-analytic strategy

All statistical analyses were conducted in R (Version 4.4.2) (R Core Team, 2024) using the metafor package (Viechtbauer, 2010).

Random-effects meta-analytic models were estimated using restricted maximum likelihood (REML) estimation because true effect sizes were expected to vary across studies owing to differences in participant characteristics, sampling procedures, study settings, measurement instruments, and cultural contexts. REML estimation is widely recommended for estimating between-study variance under random-effects assumptions because of its favorable statistical properties (Viechtbauer, 2010).

A conventional two-level random-effects model was employed, with sampling variance represented at Level 1 and between-study heterogeneity represented at Level 2. This modeling strategy was considered appropriate because the dataset contained relatively few statistically dependent effect sizes within individual studies, and each study generally contributed a single independent effect size for each psychological construct (Van den Noortgate et al., 2013).

Effect sizes were synthesized separately for each psychological correlate to preserve conceptual specificity and to avoid combining substantively distinct constructs within a single pooled estimate. When multiple eligible effect sizes representing the same construct were available within a study, a single independent estimate was retained or statistically combined using established meta-analytic procedures to minimize statistical dependence (Borenstein et al., 2009).

To examine whether pooled associations differed across categories of psychological correlates, mixed-effects omnibus moderator tests (QM statistics) were conducted within the meta-analytic framework. These analyses were intended to evaluate between-group differences in pooled effect sizes while accounting for residual between-study heterogeneity.

Because all included studies were observational, the meta-analysis synthesized correlational associations only. Consequently, all findings were interpreted descriptively, and no causal or directional inferences were drawn.

### 2.10 Heterogeneity and sensitivity analyses

Statistical heterogeneity was evaluated using Cochran's Q statistic, the I^2^ statistic, between-study variance estimates (τ^2^), and 95% prediction intervals. Cochran's Q was used to assess whether the observed variability in effect sizes exceeded that expected by sampling error alone, whereas the I^2^ statistic quantified the proportion of total observed variance attributable to between-study heterogeneity rather than chance (Higgins et al., 2003).

Consistent with current recommendations, I^2^ values were not interpreted in isolation because they are influenced by both sampling precision and between-study variance and therefore do not represent an absolute measure of heterogeneity (Borenstein et al., 2017). Accordingly, heterogeneity was interpreted by jointly considering Q statistics, I^2^ estimates, prediction intervals, and the methodological characteristics of the included studies.

Because prediction intervals provide an estimate of the range within which the true effect of a future comparable study is expected to fall, 95% prediction intervals were calculated for all primary meta-analyses whenever estimation was statistically feasible (IntHout et al., 2016; Riley et al., 2011). These intervals were used to evaluate the potential generalizability and dispersion of pooled effect estimates beyond the average summary effect.

Sensitivity analyses were conducted to evaluate the robustness of the findings. These analyses included (a) leave-one-out analyses to examine the influence of individual studies, (b) re-analysis after excluding studies with sample sizes below 50 participants, and (c) assessment of influential cases using standard influence diagnostics available within the metafor package (Viechtbauer, 2010). Studies meeting the predefined criteria for substantial statistical influence were excluded from the primary meta-analytic models, and the consistency of pooled estimates before and after sensitivity checks was considered an indicator of result robustness.

### 2.11 Moderator analyses

Exploratory moderator analyses were conducted to investigate potential sources of between-study heterogeneity when sufficient data were available**.** Consistent with commonly recommended practices in meta-analysis (Borenstein et al., 2009; Higgins et al., 2022), categorical moderators were examined only when at least 10 studies contributed to the overall analysis and a minimum of four studies were available within each subgroup. Continuous moderators were evaluated only when data from at least 10 studies were available.

Continuous moderators, including mean participant age, publication year, sample size, percentage of female participants, and reported internal consistency coefficients (Cronbach's α), were examined using mixed-effects meta-regression models. Categorical moderators, including country and timing relative to the COVID-19 pandemic, were evaluated using subgroup analyses within the mixed-effects framework.

Instrument orientation was examined as an exploratory measurement-related moderator to assess whether dysregulation-oriented and distress-oriented operationalizations of PPU were associated with variation in effect sizes across psychological correlates. These analyses were intended to evaluate measurement-related variability rather than to test causal pathways or distinct subtypes of PPU.

Because moderator analyses were based on observational study characteristics and subgroup sample sizes varied considerably across analyses, all moderator findings were interpreted as exploratory and hypothesis-generating rather than confirmatory. No causal interpretations were made.

### 2.12 Instrument orientation as a moderator

To directly examine the influence of measurement heterogeneity, additional subgroup analyses were conducted based on the a priori conceptual classification of PPU instruments as either behavioral dysregulation-oriented or distress-oriented measures.

For psychological correlates with sufficient data, pooled effect sizes were estimated separately within each measurement category using random-effects subgroup models within a mixed-effects framework. Differences between subgroup estimates were evaluated using omnibus QM tests for moderators within the meta-analytic framework.

These analyses were designed to determine whether variation in measurement emphasis was associated with differences in observed effect sizes across studies. Importantly, the analyses were not intended to identify distinct etiological forms of PPU, but rather to evaluate whether differences in operationalization contributed to variability in reported psychological correlates.

Because several instruments incorporate elements of both behavioral dysregulation and subjective distress, and because some subgroup analyses included relatively small numbers of studies, results were interpreted cautiously and regarded as exploratory.

### 2.13 Assessment of publication bias

Potential publication bias and small-study effects were evaluated using visual inspection of funnel plots, Egger's regression test (Egger et al., 1997), and the trim-and-fill procedure (Duval & Tweedie, 2000), when statistically feasible.

Visual inspection of funnel plots was used to evaluate the symmetry of effect size distributions. Egger's regression test was applied to statistically assess funnel plot asymmetry, with significant asymmetry interpreted as a possible indicator of small-study effects rather than definitive evidence of publication bias.

To evaluate the potential impact of unpublished or missing studies, Duval and Tweedie's trim-and-fill procedure was applied to estimate the number of potentially missing studies and to calculate adjusted pooled effect sizes.

Because several psychological correlates were represented by relatively small numbers of studies, publication bias analyses were interpreted cautiously and regarded as exploratory when the number of available studies was limited.

Funnel plots illustrating the primary analyses are presented in Fig. 3, Fig. 4.

## 3 Results

### 3.1 Characteristics of the included studies

The characteristics of the included studies are summarized in Table 1, with additional information provided in Table S1 (Supplementary Materials).

**Table 1:** Study characteristic.

Variable		k	N	Mean_percent
Independent studies/samples		111	121,728	100
Publication date (2012–2025)		111	121,728	
	2012–2016	13	18,221	14.96%
	2017–2021	53	59,213	48.64%
	2022–2025	45	44,294	36.38%
% female		107	119,764	45.90%
Age		95	85,095	27.5
% married		61	57,019	98.97%
% employed		15	9719	58.40%
sexual orientation				
	heterosexual	47	51,402	84.80%
Education level				
	University degree %	59	61,178	65.40%
religion				
	Christian	36	33,100	53.70%
	Jewish	15	16,731	7.84%
	Muslim	19	27,503	15.50%
	Hindu	17	25,849	4.06%
	Buddhist	13	12,647	1.75%
	Atheist/Agnostic	24	20,934	29.70%
ethnicity				
	white/Caucasian	42	36,364	73.60%
	black/African American	40	36,064	9.78%
	Latino/Hispanic	36	34,064	7.29%
	Asian/Pacific-Islander	38	35,608	13.70%
	American Indian/Alaska Native	15	15,198	2%
	Middle Eastern	14	15,241	3.86%
	Other	30	26,031	4.61%
COVID-19				
	during	44	37,993	31.20%
	post	33	45,125	37.10%
	pre	34	38,610	31.70%
Data collection method				
	in-person	3	866	0.72%
	mixed	2	244	0.20%
	online	97	118,590	99.10%

*Note:*k = Number of studies/samples contributing available data; N = number of participants. Percentages may exceed 100% due to incomplete demographic.

The included studies were published between 2012 and 2025. When weighted by sample size, 14.9% of participants were drawn from studies published between 2012 and 2016, 48.6% from 2017 to 2021, and 36.4% from 2022 to 2025. Across all eligible studies, the cumulative sample comprised 121,728 participants.

Educational attainment was reported in 59 studies, among which 65.4% of participants had completed university education. Religious affiliation was reported less consistently; among studies providing these data, 53.7% of participants identified as Christian, 29.7% as atheist or agnostic, and 15.5% as Muslim.

Age information was available for 95 studies. Of these, 53 primarily included adult samples (26–55 years), whereas 34 focused on young adults (18–25 years). The sample-size-weighted mean participant age across studies was 27.5 years. Because age variability (standard deviations) was inconsistently reported, pooled estimates of age variability were not calculated.

Across studies reporting gender composition, the mean proportion of female participants was 45.9%. Ethnicity was reported in a subset of studies; among those reporting these data, 73.6% of participants were White/Caucasian, 13.7% Asian/Pacific Islander, and 9.8% Black/African American. Regarding the timing of data collection relative to the COVID-19 pandemic, 31.7% of studies were conducted before the pandemic, 31.2% during the pandemic, and 37.1% after the pandemic.

### 3.2 Outlier detection and sensitivity analyses

Potential outlying and influential studies were evaluated using standardized residuals, Cook's distance, and leave-one-out influence diagnostics.

One study met the predefined criteria for substantial statistical influence and was excluded from subsequent meta-analytic models. No additional studies demonstrated disproportionate influence on pooled effect estimates.

Sensitivity analyses, including leave-one-out procedures and exclusion of studies with sample sizes below 50 participants, produced results that were highly consistent with the primary analyses. Although small changes in pooled effect sizes were observed for several constructs, the overall pattern of associations remained unchanged.

### 3.3 Pooled associations between psychological correlates and PPU

**Fig. 2:**
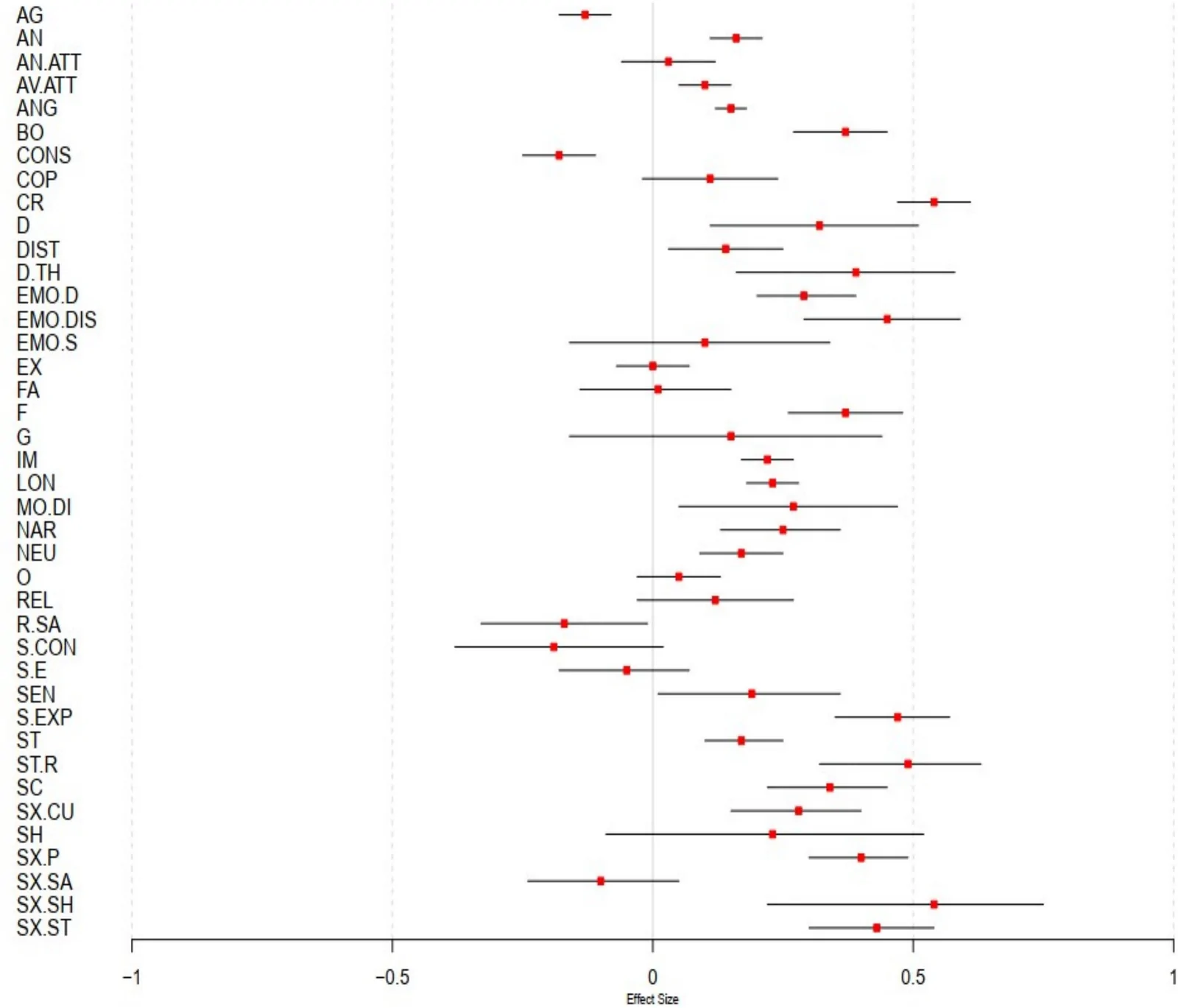
Forest plot of effect sizes (Pearson's r) with 95% confidence intervals.

The pooled meta-analytic estimates are presented in Table 2, and corresponding forest plots are shown in Fig. 2. The estimates reported in Table 2 reflect the primary meta-analytic models after influence diagnostics.

**Table 2:** Meta-analysis of all psychological constructs associated with PPU.

Fundamental	k	N	r	CI_lower	CI_upper	Z_value	P_value	Q	*I*^*2*^*(%)*	*τ*^*2*^	PI-lower	PI-upper
AG	8	3171	−0.13	−0.18	−0.08	−5.22***	0.00	11.76	40.46	0.00	−0.21	−0.05
AN	16	8335	0.16	0.11	0.21	6.25***	0.00	75.55	80.15	0.05	−0.14	0.46
AN.ATT	9	10,695	0.03	−0.06	0.12	0.56	0.57	144.88	94.48	0.14	−0.71	0.77
AV.ATT	7	4069	0.10	0.05	0.15	4.08***	0.00	12.35	51.40	0.02	−0.18	0.38
ANG	4	5203	0.15	0.12	0.18	10.82***	0.00	1.20	0.00	0.00	0.12	0.18
BO	5	3969	0.37	0.27	0.45	6.95***	0.00	46.41	91.38	0.10	−0.27	0.82
CONS	8	3171	−0.18	−0.25	−0.11	−4.73***	0.00	27.46	74.51	0.03	−0.49	0.13
COP	11	6085	0.11	−0.02	0.24	1.67	0.09	231.46	95.68	0.18	−0.73	0.95
CR	4	3690	0.54	0.47	0.61	12.03***	0.00	23.43	87.19	0.07	0.05	0.82
D	17	20,921	0.32	0.11	0.51	2.95***	0.00	3390.29	99.53	0.30	−0.79	0.93
DIST	7	3162	0.14	0.03	0.25	2.48*	0.01	53.46	88.78	0.08	−0.45	0.65
D.TH	4	1675	0.39	0.16	0.58	3.2***	0.00	68.87	95.64	0.18	−0.59	0.90
EMO·D	12	4261	0.29	0.20	0.39	5.72***	0.00	119.03	90.76	0.10	−0.25	0.72
EMO.DIS	5	3969	0.45	0.29	0.59	5.12***	0.00	138.56	97.11	0.22	−0.53	0.93
EMO·S	4	790	0.10	−0.16	0.34	0.73	0.46	39.75	92.45	0.12	−0.63	0.72
EX	9	3307	0.00	−0.07	0.07	0.03	0.98	28.42	71.85	0.03	−0.31	0.31
FA	10	4409	0.01	−0.14	0.15	0.08	0.94	204.65	95.60	0.17	−0.82	0.84
F	4	3068	0.37	0.26	0.48	5.89***	0.00	38.61	92.23	0.12	−0.21	0.78
G	5	1631	0.15	−0.16	0.44	0.98	0.33	149.45	97.32	0.24	−0.86	0.93
IM	21	33,476	0.22	0.17	0.27	8.01***	0.00	389.79	94.87	0.15	−0.54	0.77
LON	21	19,110	0.23	0.18	0.28	8.47***	0.00	235.60	91.51	0.11	−0.36	0.73
MO.DI	17	14,686	0.27	0.05	0.47	2.44*	0.01	2862.49	99.44	0.29	−0.84	0.91
NAR	6	1903	0.25	0.13	0.36	4.02***	0.00	32.79	84.75	0.06	−0.17	0.59
NEU	13	8205	0.17	0.09	0.25	4.01***	0.00	169.65	92.93	0.13	−0.39	0.66
O	7	2518	0.05	−0.03	0.13	1.25	0.21	20.10	70.15	0.03	−0.26	0.36
REL	18	18,994	0.12	−0.03	0.27	1.56	0.12	1813.74	99.06	0.27	−0.90	0.92
R.SA	6	26,230	−0.17	−0.33	−0.01	−2.09*	0.04	572.15	99.13	0.28	−0.91	0.57
S.CON	7	8512	−0.19	−0.38	0.02	−1.79	0.07	468.20	98.72	0.26	−0.98	0.63
S.E	5	5255	−0.05	−0.18	0.07	−0.87	0.39	61.22	93.47	0.14	−0.60	0.50
SEN	4	3951	0.19	0.01	0.36	2.09*	0.04	44.15	93.20	0.13	−0.47	0.73
S.EXP	4	3068	0.47	0.35	0.57	7.15***	0.00	43.23	93.06	0.13	−0.10	0.82
ST	13	6463	0.17	0.10	0.25	4.34***	0.00	109.30	89.02	0.09	−0.35	0.61
ST.R	5	3969	0.49	0.32	0.63	5.16***	0.00	167.10	97.61	0.23	−0.47	0.96
SC	4	2400	0.34	0.22	0.45	5.49***	0.00	28.09	89.32	0.09	−0.10	0.69
SX.CU	6	3548	0.28	0.15	0.40	4.21***	0.00	75.78	93.40	0.14	−0.32	0.73
SH	4	1014	0.23	−0.09	0.52	1.40	0.16	78.82	96.19	0.19	−0.75	0.89
SX.P	6	3548	0.40	0.30	0.49	7.34***	0.00	51.39	90.27	0.10	−0.06	0.73
SX.SA	5	2247	−0.10	−0.24	0.05	−1.27	0.20	44.24	90.96	0.10	−0.52	0.32
SX.SH	5	1816	0.54	0.22	0.75	3.12***	0.00	235.98	98.30	0.25	−0.64	0.98
SX.ST	4	3068	0.43	0.30	0.54	6.17***	0.00	48.61	93.83	0.15	−0.26	0.86

Omnibus test across correlate domain qbetween(df = 39) = 209.241 *p* < 0.001.

*Note.*k = number of studies; N = number of participants; r = mean weighted effect size; CI = 95% confidence interval; z = z value of the significance test; Q = ratio of variation across studies; I2 = proportion of total observed variation attributable to between-study effect, τ^2^ = Absolute between-study variance, PI = 95% Prediction Interval. AG = agreeable, AN = Anxiety, AN.ATT = Anxiety attachment, AV.ATT = Avoidance attachment, ANG = anger, BO=Boredom Avoidance, CONS = conscientiousness, COP=Coping, CR = Craving,D=Depression, DIST = Distress, D.TH=Desire thinking, EMO·D = Emotion dysregulation, EMO.DIS = Emotional Distraction, EMO·S = Emotional stability, EX = extraversion, FA = family environment, F = fantasy, G = Guilt,IM = Impulsivity, LON = Loneliness, MO.DI = moral disapproval, NAR = Narcissism, NEU=Neuroticism, O=Openness, REL = religiousness, R.SA = Relationship satisfaction, S.CON=Self-Control, S.E = Self-esteem, SEN=Sensation seeking, S.EXP=Self Exploration, ST = Stress, ST.R = Stress Reduction, SC=Scrupulosity,SX.CU=Sexual curiosity, SH=Shame, SX.P=Sexual pleasure, SX.SA = Sexual Satisfaction, SX.SH = sexual shame, SX.ST = Lack of sexual satisfaction, *p < 0.05. **p < 0.01. ***p < 0.001.

Across psychological correlates, pooled effect sizes ranged from negligible to moderate-to-large in magnitude. The strongest pooled associations were observed for craving and sexual shame, whereas the weakest associations were observed for extraversion and family environment.

Mixed-effects omnibus analyses indicated significant differences in pooled effect sizes across psychological correlate domains, QM(39) = 209.24, *p* < 0.001, indicating that the magnitude of associations varied across constructs.

Substantial between-study heterogeneity was observed for many analyses, with I^2^ values frequently exceeding 75%. Prediction intervals were generally wider than the corresponding confidence intervals and, for several correlates, included values close to zero, indicating considerable variability in expected effect sizes across future studies and populations.

Overall, pooled correlations should therefore be interpreted as average summary estimates rather than effects expected to be identical across all study settings.

### 3.4 Conceptualization and measurement heterogeneity

Additional subgroup analyses examined whether pooled associations differed according to the dominant conceptual emphasis of the PPU measurement instruments. A comparative overview of instrument orientations is provided in Table 7.

Descriptively, studies employing behavioral dysregulation-oriented instruments generally reported stronger associations with craving, impulsivity, deficient self-control, and compulsive engagement. In contrast, studies using distress-oriented instruments tended to report relatively stronger associations with shame, guilt, moral incongruence, and religiousness.

Despite these descriptive differences, substantial overlap in correlational patterns was observed across instrument categories. Overall, the findings suggest that differences in measurement emphasis may have contributed to variation in observed effect sizes across several psychological correlates.

### 3.5 Moderator analyses

#### 3.5.1 Continuous moderators

Continuous moderator analyses were conducted when at least ten studies contributed data to a given association.Moderators included publication year, sample size, mean participant age, percentage of female participants, and reported internal consistency coefficients (Cronbach's α).Most continuous moderators were not statistically significant. Regression coefficients were generally small, and confidence intervals frequently included zero.

Several moderator effects reached nominal statistical significance in individual analyses. However, these effects were not consistently observed across psychological constructs and should therefore be interpreted cautiously.Detailed results of continuous moderator analyses are presented in Table 3. Overall, the observed associations between psychological correlates and PPU were broadly comparable across the study characteristics examined.

**Table 3:** Continuous moderator results.

Variable	Moderator	b	SE	CI_Lower	CI_Upper	Z Value	QM	T^2^	k
Anxiety									
	N	4.90 × 10^−5^	9.80 × 10^−5^	−1.43 × 10^−4^	2.40 × 10^−4^	0.50	0.25	0.01	16
	year	0.01	0.01	−0.01	0.02	0.77	0.59	0.01	16
	mean age	−0.01	0.01	−0.02	0.00	−1.41	1.99	0.01	13
	%female	0.30	0.19	−0.07	0.68	1.59	2.53	0.01	14
	Anxiety_instrument_α	−0.33	0.59	−1.49	0.82	−0.57	0.32	0.01	15
	porno_instrument_α	0.27	0.35	−0.41	0.95	0.77	0.60	0.01	11

Coping									
	N	−1.20 × 10^−4^	9.90 × 10^−5^	−3.13 × 10^−4^	7.30 × 10^−5^	−1.21	1.47	0.03	11
	year	−0.01	0.03	−0.08	0.06	−0.32	0.10	0.04	11
	mean age	−0.02	0.02	−0.05	0.02	−0.81	0.66	0.03	10
	%female	0.52	0.22	0.09	0.96	2.36*	5.58	0.02	11

Depression									
	N	6.90 × 10^−5^	2.70 × 10^−5^	1.30 × 10^−5^	1.25 × 10^−4^	2.41*	5.80	0.07	17
	year	0.03	0.02	−0.01	0.08	1.48	2.19	0.08	17
	mean age	−0.01	0.01	−0.04	0.02	−0.66	0.43	0.10	15
	%female	−0.52	0.74	−1.98	0.93	−0.70	0.49	0.10	16
	Depression_instrument_α	−1.08	1.77	−4.55	2.39	−0.61	0.37	0.09	17
	porno_instrument_α	−0.88	1.75	−4.30	2.54	−0.50	0.25	0.11	14
	porno_mean	0.05	0.04	−0.04	0.14	1.15	1.32	0.07	11

Emotion dysregulation									
	N	−1.97 × 10^−4^	2.74 × 10^−4^	−7.33 × 10^−4^	3.40 × 10^−4^	−0.72	0.51	0.06	12
	year	−0.03	0.03	−0.09	0.03	−0.98	0.96	0.06	12
	mean age	0.01	0.01	−0.01	0.02	0.68	0.46	0.06	12
	%female	−0.12	0.24	−0.60	0.35	−0.51	0.26	0.06	12
	Emotion dysregulation_instrument_α	1.86	0.65	0.60	3.13	2.89**	8.33	0.03	12
	porno_instrument_α	3.37	1.93	−0.40	7.15	1.75	3.06	0.06	10

Family environment									
	N	−2.62 × 10^−4^	1.97 × 10^−4^	−6.48 × 10^−4^	1.24 × 10^−4^	−1.33	1.77	0.04	10
	year	−7.47 × 10^−4^	3.11 × 10^−4^	−1.36 × 10^−3^	−1.38 × 10^−4^	−2.41*	5.79	0.03	10

Impulsivity									
	N	−1.04 × 10^−5^	1.61 × 10^−5^	−4.20 × 10^−5^	2.11 × 10^−5^	−0.65	0.42	0.04	21
	year	0.01	0.02	−0.04	0.05	0.23	0.05	0.05	21
	mean age	0.01	0.01	0.00	0.03	1.69	2.86	0.04	21
	%female	−0.11	0.18	−0.45	0.24	−0.60	0.36	0.05	21
	Impulsivity_instrument_α	2.19	0.90	0.42	3.95	2.42*	5.88	0.04	20
	porno_instrument_α	1.64	1.25	−0.82	4.09	1.31	1.71	0.04	20
	porno_mean	0.05	0.04	−0.04	0.13	1.02	1.05	0.07	14

Loneliness									
	N	−1.62 × 10^−5^	2.91 × 10^−5^	−7.32 × 10^−5^	4.08 × 10^−5^	−0.56	0.31	0.03	21
	year	−6.79× 10^−4^	1.34× 10^−4^	−9.40× 10^−4^	−4.17× 10^−4^	−5.08***	25.84	0.01	21
	mean age	1.25× 10^−3^	0.01	−0.01	0.01	0.24	0.06	0.01	13
	%female	−0.02	0.12	−0.25	0.21	−0.18	0.03	0.01	19
	Loneliness_instrument_α	−0.31	0.60	−1.49	0.86	−0.52	0.27	0.03	19
	porno_instrument_α	0.85	0.98	−1.08	2.77	0.86	0.75	0.03	16
	porno_mean	0.00	0.02	−0.03	0.03	−0.02	0.00	0.01	13

Moral disapproval									
	N	5.15 × 10^−5^	1.07 × 10^−4^	−1.58 × 10^−4^	2.61 × 10^−4^	0.48	0.23	0.17	17
	year	0.02	0.06	−0.09	0.12	0.27	0.07	0.17	17
	mean age	−0.01	0.01	−0.03	0.02	−0.46	0.21	0.16	14
	%female	0.28	0.54	−0.77	1.33	0.52	0.27	0.17	17
	moral disapproval_instrument_α	−1.77	4.08	−9.77	6.23	−0.43	0.19	0.17	15

Neuroticism									
	N	−1.89 × 10^−4^	9.84 × 10^−5^	−3.82 × 10^−4^	3.01 × 10^−6^	−1.93	3.72	0.02	13
	year	−3.44 × 10^−4^	1.90 × 10^−4^	−7.17 × 10^−4^	2.93 × 10^−5^	−1.81	3.26	0.02	13
	mean age	4.08 × 10^−3^	0.01	−0.01	0.02	0.46	0.21	0.02	12
	%female	−0.10	0.18	−0.44	0.24	−0.57	0.32	0.02	13
	Neuroticism_instrument_α	1.02	1.04	−1.03	3.06	0.97	0.95	0.02	10

Religiousness									
	N	2.84 × 10^−5^	7.22 × 10^−5^	−1.13 × 10^−4^	1.70 × 10^−4^	0.39	0.16	0.09	18
	year	0.03	0.03	−0.03	0.08	1.01	1.03	0.09	18
	mean age	0.30	0.14	0.03	0.56	2.18*	4.75	0.01	16
	%female	−0.14	0.28	−0.69	0.41	−0.51	0.26	0.09	17
	Religiousness_instrument_α	−1.21	1.59	−4.32	1.90	−0.76	0.58	0.09	17

Stress									
	N	−2.35 × 10^−4^	1.13 × 10^−4^	−4.58 × 10^−4^	−1.38 × 10^−5^	−2.08*	4.34	0.02	13
	year	0.02	0.01	−0.01	0.04	1.38	1.90	0.02	13
	mean age	0.01	0.01	0.00	0.03	1.46	2.13	0.02	13
	%female	−0.52	0.22	−0.96	−0.09	−2.35*	5.53	0.02	13
	Stress_instrument_α	−0.12	0.93	−1.94	1.70	−0.13	0.02	0.02	12
	porno_instrument_α	0.55	1.10	−1.60	2.70	0.50	0.25	0.03	12
	porno_mean	0.08	0.05	−0.03	0.18	1.42	2.01	0.02	10

Note: Q_M_ = Test of moderators; T^2^ = between-study variance estimate, b = regression coefficient; SE = standard error; CI = confidence interval; z = z value of the significance test; k = number of samples,*p < 0.05. **p < 0.01. ***p < 0.001.

#### 3.5.2 Categorical moderators

Categorical moderator analyses examined differences according to country, timing of data collection relative to the COVID-19 pandemic, and PPU instrument type.

Most subgroup comparisons were not statistically significant.

Significant moderator effects were observed for selected constructs. COVID-19 timing significantly moderated associations involving stress, whereas instrument type significantly moderated associations involving loneliness, neuroticism, and religiousness.

Residual heterogeneity remained substantial in most subgroup analyses, indicating that additional methodological or conceptual factors likely contributed to between-study variability.

Detailed subgroup results are presented in Table 4.

**Table 4:** Categorical moderator results.

Variables	Moderator	k	N	r	r_ci.lb	r_ci.ub	Q	I2
Anxiety								
	COVID19	(Q = 1.76, df = 1, *p* = 0.18)						
	pre	6	4634	0.10	0.03	0.17	38.02	75.33
	during	7	2599	0.17	0.10	0.23	38.02	75.33
	**PPU instrument** **type**	(Q = 0.00, df = 1, *p* = 0.98)						
	Dysregulation-oriented	6	2324	0.19	0.10	0.28	46.16	83.76
	Distress-oriented	5	3661	0.19	0.10	0.28	46.16	83.76

Coping								
	COVID19	(Q = 1.69, df = 1, *p* = 0.19)						
	post	4	597	0.16	0.02	0.30	228.01	94.52
	during	7	5488	0.00	−0.20	0.20	228.01	94.52

Depression								
	COVID19	(Q = 2.56, df = 2, *p* = 0.28)						
	pre	6	9299	0.18	−0.06	0.39	705.24	98.22
	during	7	2117	0.39	0.19	0.57	705.24	98.22
	post	4	9505	0.41	0.13	0.62	705.24	98.22
	**PPU instrument** **type**	(Q = 0.68, df = 1, *p* = 0.41)						
	Dysregulation-oriented	8	15,907	0.28	0.03	0.50	2578.51	99.06
	Distress-oriented	6	3803	0.41	0.21	0.58	2578.51	99.06

Family environment								
	**PPU instrument** **type**	(Q = 0.27, df = 1, *p* = 0.61)						
	Dysregulation-oriented	4	3203	0.039	−0.153	0.227	187.758	95.593
	Distress-oriented	6	1206	−0.040	−0.263	0.186	187.758	95.593

Impulsivity								
	Country	(Q = 0.09, df = 1, *p* = 0.77)						
	China	5	3500	0.20	0.01	0.37	260.60	97.16
	Germany	6	4800	0.24	0.04	0.41	260.60	97.16
	COVID19	(Q = 0.15, df = 1, *p* = 0.70)						
	post	6	1212	0.25	0.12	0.37	370.54	98.14
	during	12	23,072	0.20	0.01	0.38	370.54	98.14

Loneliness								
	COVID19	(Q = 1.18, df = 2, *p* = 0.56)						
	pre	5	12,744	0.17	0.02	0.30	207.46	95.59
	during	6	2748	0.22	0.09	0.35	207.46	95.59
	post	10	3618	0.26	0.16	0.36	207.46	95.59
	PPU**instrument** **type**	(Q = 4.69, df = 1, *p* = 0.03)						
	Dysregulation-oriented	12	8825	0.12	−0.03	0.26	146.11	93.27
	Distress-oriented	4	6077	0.30	0.22	0.38	146.11	93.27

Moral disapproval								
	COVID19	(Q = 0.15, df = 1, *p* = 0.70)						
	pre	4	7324	0.23	−0.02	0.46	2231.18	99.33
	during	11	6704	0.32	−0.10	0.64	2231.18	99.33

Neuroticism								
	**PPU instrument** **type**	(Q = 14.22, df = 1, *p* = 0.00)						
	Dysregulation-oriented	4	1870	0.27	0.23	0.32	14.64	34.96
	Distress-oriented	6	3267	0.13	0.08	0.19	14.64	34.96

Religiousness								
	COVID19	(Q = 0.59, df = 2, *p* = 0.74)						
	pre	7	11,316	0.06	−0.17	0.28	1764.80	98.93
	during	7	5163	0.18	−0.05	0.40	1764.80	98.93
	post	4	2515	0.12	−0.19	0.40	1764.80	98.93
	**PPU instrument** **type**	(Q = 4.45, df = 1, *p* = 0.04)						
	Dysregulation-oriented	4	2515	0.44	0.25	0.60	262.10	97.90
	Distress-oriented	5	6327	0.12	−0.13	0.35	262.10	97.90

Stress								
	COVID19	(Q = 4.88, df = 1, *p* = 0.03)						
	pre	4	3398	0.28	0.18	0.37	31.81	83.09
	post	6	1590	0.12	0.01	0.22	31.81	83.09
	PPU instrument type	(Q = 0.61, df = 1, *p* = 0.44)						
	Dysregulation-oriented	7	3370	0.13	−0.05	0.29	101.41	93.11
	Distress-oriented	4	2695	0.21	0.08	0.33	101.41	93.11

Note. k = number of studies/samples; N = number of participants; r = mean weighted effect size; CI = confidence interval; Q = ratio of variation to within-study error; *I*^*2*^ = proportion of total observed variation attributable to between-study effects. *p < 0.05. **p < 0.01. ***p < 0.001.

#### 3.5.3 Publication bias

Potential publication bias was evaluated using visual inspection of funnel plots, Egger's regression test, and the trim-and-fill procedure.

**Fig. 3:**
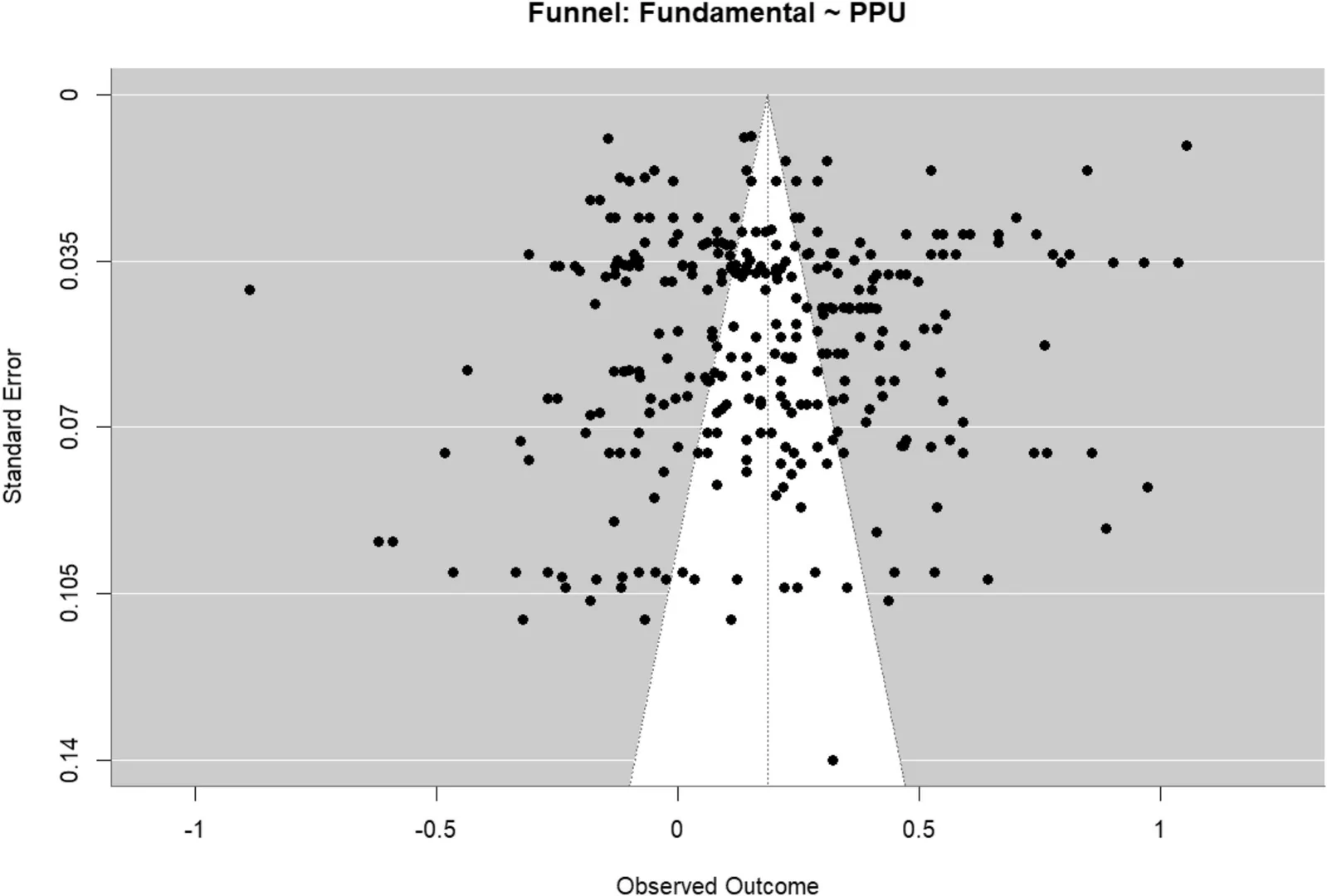
Funnel plot of effect sizes for all included studies in the meta-analysis.

**Fig. 4:**
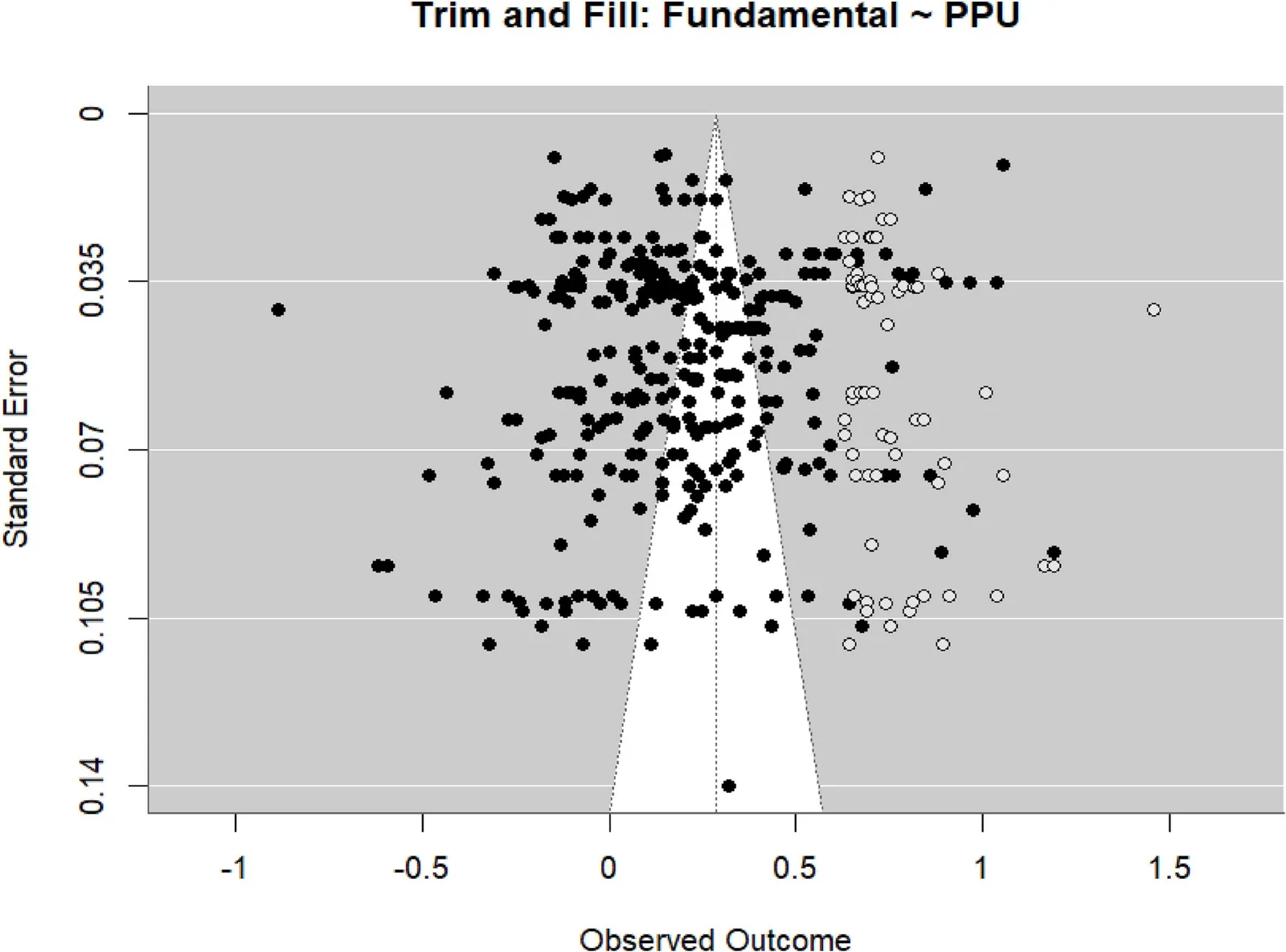
Trim and Fill Funnel plot.

Visual inspection of the funnel plots (Fig. 3, Fig. 4) suggested generally modest asymmetry in some analyses.

Trim-and-fill analyses identified potentially missing studies for several psychological correlates, including anxiety, depression, desire thinking, loneliness, and religiousness. Adjustment for these potentially missing studies resulted in only modest changes in pooled effect sizes.

For most remaining psychological correlates, there was limited evidence of funnel plot asymmetry or trim-and-fill imputation. However, because several analyses included relatively small numbers of studies, publication bias results should be interpreted cautiously.

Overall, trim-and-fill adjustments produced only minor attenuation of pooled correlations and did not materially alter the overall pattern of findings. Detailed publication bias analyses are presented in Table 5. (See Table 6.)

**Table 5:** Results of publication bias analyses.

Variable	k	Obs_r	CI_low	CI_high	Imputed studies(Direction)	Adj_r	Adj_ CI _low	Adj_ CI _high	Eggers *z*
AG	8	−0.13	−0.18	−0.08	0	−0.13	−0.18	−0.08	−2.57
AN	16	0.16	0.11	0.21	1(R)	0.17	0.11	0.23	−0.82
AN.ATT	9	0.02	−0.08	0.12	0	0.02	−0.08	0.12	−1.00
AV.ATT	7	0.10	0.05	0.15	0	0.10	0.05	0.15	−0.13
ANG	4	0.15	0.12	0.18	0	0.15	0.12	0.18	0.11
BO	5	0.38	0.27	0.49	0	0.38	0.27	0.49	−1.79
CONS	8	−0.18	−0.26	−0.10	0	−0.18	−0.26	−0.10	−2.11
COP	11	0.11	−0.01	0.23	0	0.11	−0.01	0.23	0.45
CR	4	0.54	0.48	0.72	0	0.60	0.48	0.72	−4.84
D	17	0.33	0.19	0.48	3(R)	0.39	0.25	0.53	−0.59
DIST	7	0.14	0.01	0.27	0	0.14	0.01	0.27	−2.03
D.TH	4	0.41	0.20	0.62	1(R)	0.36	0.17	0.55	1.95
EMO·D	12	0.30	0.16	0.44	0	0.30	0.16	0.44	0.25
EMO.DIS	5	0.49	0.31	0.66	0	0.49	0.31	0.66	−0.90
EMO·S	4	0.10	−0.19	0.39	0	0.10	−0.19	0.39	0.82
EX	9	0.00	−0.09	0.09	0	0.00	−0.09	0.09	0.43
FA	10	0.01	−0.14	0.15	0	0.01	−0.14	0.15	1.19
F	4	0.39	0.28	0.51	0	0.39	0.28	0.51	−0.44
G	5	0.16	−0.19	0.50	0	0.16	−0.19	0.50	−1.16
IM	21	0.23	0.14	0.32	0	0.23	0.14	0.32	1.55
LON	21	0.23	0.16	0.30	5(R)	0.28	0.21	0.36	−0.05
MO.DI	17	0.28	0.09	0.47	0	0.28	0.09	0.47	0.44
NAR	6	0.26	0.11	0.41	0	0.26	0.11	0.41	0.39
NEU	13	0.17	0.09	0.26	0	0.17	0.09	0.26	2.40
O	7	0.05	−0.03	0.13	0	0.05	−0.03	0.13	−0.46
REL	18	0.12	−0.02	0.26	3(R)	0.18	0.05	0.32	−0.02
R.SA	6	−0.18	−0.40	0.03	0	−0.18	−0.40	0.03	−3.19
S.CON	7	−0.19	−0.46	0.08	0	−0.19	−0.46	0.08	−0.34
S.E	5	−0.05	−0.21	0.11	0	−0.05	−0.21	0.11	1.26
SEN	4	0.18	−0.08	0.43	0	0.18	−0.08	0.43	−2.32
S.EXP	4	0.50	0.37	0.64	0	0.50	0.37	0.64	−6.56
ST	13	0.18	0.09	0.26	0	0.18	0.09	0.26	2.62
ST.R	5	0.54	0.34	0.73	0	0.54	0.34	0.73	−1.29
SC	4	0.35	0.21	0.50	0	0.35	0.21	0.50	2.19
SX.CU	6	0.28	0.10	0.47	0	0.28	0.10	0.47	−6.16
SH	4	0.24	−0.09	0.56	0	0.24	−0.09	0.56	−1.31
SX.P	6	0.42	0.31	0.53	0	0.42	0.31	0.53	−0.63
SX.SA	5	−0.11	−0.35	0.13	0	−0.11	−0.35	0.13	−2.51
SX.SH	5	0.54	0.29	0.91	0	0.60	0.29	0.91	−0.40
SX.ST	4	0.46	0.31	0.61	0	0.46	0.31	0.61	−6.31

**Predictor**					**b**	**SE**	**z**	**p**	**95% CI**
Intercept (Distress + reference instrument)					0.265	0.035	7.7	< 0.001	[0.198, 0.333]
Process (Dysregulation vs Distress)					0.021	0.054	0.38	0.703	[−0.085, 0.126]
Instrument (Dysregulation-oriented vs Distress-oriented)					−0.026	0.047	−0.55	0.581	[−0.119, 0.067]

Note. Obs. r = observed average effect size; Adj. r = adjusted average effect size. CI = confidence interval; L = Left; R = Right. AG = agreeable, AN = Anxiety,AN.ATT = Anxiety attachment, AV.ATT = Avoidance attachment, ANG = anger, BO=Boredom Avoidance, CONS = conscientiousness, COP=Coping, CR = Craving,D=Depression, DIST = Distress, D.TH=Desire thinking,EMO·D = Emotion dysregulation, EMO.DIS = Emotional Distraction, EMO·S = Emotional stability, EX = extraversion, FA = family environment, F = fantasy, G = Guilt,IM = Impulsivity, LON = Loneliness, MO.DI = moral disapproval, NAR = Narcissism, NEU=Neuroticism, O=Openness, REL = religiousness, R.SA = Relationship satisfaction, S.CON=Self-Control, S.E = Self-esteem, SEN=Sensation seeking,S.EXP=Self Exploration, ST = Stress, ST.R = Stress Reduction, SC=Scrupulosity,SX.CU=Sexual curiosity, SH=Shame, SX.P=Sexual pleasure, SX.SA = Sexual Satisfaction, SX.SH = sexual shame, SX.ST = Lack of sexual satisfaction.

Note: Because the number of studies within these sub-analyses was small (k < 10), publication bias tests are likely to be underpowered and therefore should be interpreted with caution as exploratory rather than definitive evidence regarding small-study effects or reporting bias.

**Table 7:** Comparison of PPU measurement orientations and summary of associated findings.

Conceptualization (theoretical frame)	Core definition	Typical operationalization (example measures)	Key distinctive correlates	Summary of findings
Regulation-oriented conceptualizations	PPU is conceptualized primarily as impaired control over pornography use, characterized by craving, compulsive engagement, salience, persistence despite adverse consequences, and functional impairment.	PPCS, PPCS-6, BPS, s-IAT-sex / s-IAT-porn, PPUS, and HBI when pornography-specific data were extracted. This category also includes addiction- or CSBD-oriented operationalizations when the dominant emphasis was behavioral dysregulation.	Craving, impulsivity, deficient self-control, emotion dysregulation, compulsive engagement, and other self-regulatory variables.	Studies using dysregulation-oriented measures tended to show relatively stronger associations with craving, impulsivity, deficient self-control, and emotion regulation difficulties. However, prediction intervals were often wide, indicating substantial variability across samples and contexts.
Distress-oriented conceptualizations	PPU is conceptualized primarily as subjective distress or perceived problematic use related to guilt, shame, moral incongruence, negative self-evaluation, or value-based conflict.	CPUI / CPUI-9, self-perceived pornography addiction items, moral incongruence measures, and distress-, guilt-, or shame-oriented subscales. This category also includes PPMI- or general-distress operationalizations when the dominant emphasis was evaluative distress.	sexual shame, guilt, moral disapproval, religiosity, scrupulosity, negative self-evaluation, and moral conflict.	studies using distress-oriented measures tended to show relatively stronger associations with shame, guilt, moral incongruence, religiousness, and related evaluative distress variables. Nevertheless, substantial overlap with dysregulation-related correlates was observed, suggesting that these categories should not be interpreted as mutually exclusive subtypes.

**Note.** PPU = problematic pornography use; PPCS = Problematic Pornography Consumption Scale; BPS = Brief Pornography Screen; s-IAT-sex/s-IAT-porn = Short Internet Addiction Test adapted for online sexual activities or pornography use; PPUS = Problematic Pornography Use Scale; HBI = Hypersexual Behavior Inventory; CPUI = Cyber Pornography Use Inventory; PPMI = Pornography Problems due to Moral Incongruence. This classification is intended as a heuristic conceptual framework and does not imply mutually exclusive dimensions of PPU. Several instruments contain overlapping dysregulation- and distress-related content; therefore, classifications were based on the dominant conceptual emphasis of each measure rather than on the assumption of discrete subtype.

**Table 6:** Meta-regression results for process and instrument type moderators.

**Predictor**	**b**	**SE**	**z**	**p**	**95% CI**
Intercept (Distress + reference instrument)	0.265	0.035	7.7	<.001	[0.198, 0.333]
Process (Dysregulation vs Distress)	0.021	0.054	0.38	0.703	[-0.085, 0.126]
Instrument (Dysregulation-oriented vs Distress-oriented)	-0.026	0.047	-0.55	0.581	[-0.119, 0.067]

QM (2) = 0.33, *p* = 0.847.

QE (136) = 10,091.18, *p* < 0.001.

Note: Q_M_ = Test of moderators, b = regression coefficient; SE = standard error; CI = confidence interval; z = z value of the significance test; *p < 0.05. **p < 0.01. ***p < 0.001.

## 4 Discussion

### 4.1 Overview of findings

This meta-analysis synthesized evidence from 97 independent studies to examine the psychological correlates of problematic pornography use (PPU), with particular attention to differences in how PPU has been conceptualized and measured across the literature. Rather than treating PPU as a single homogeneous construct, the present review adopted a measurement-informed perspective, recognizing that commonly used assessment instruments differ in their conceptual emphasis and therefore may capture partially different aspects of problematic pornography-related experiences. This perspective extends previous quantitative syntheses by explicitly considering the potential influence of measurement heterogeneity on observed associations.

Overall, the findings indicate that PPU is associated with a broad range of psychological variables spanning domains related to behavioral self-regulation, emotional functioning, interpersonal processes, and evaluative distress. Importantly, however, the strength of these associations varied significantly across psychological correlate domains, as indicated by omnibus mixed-effects analyses, suggesting that psychological correlates are not uniformly related to PPU. These results support the view that PPU is best understood as a multidimensional and heterogeneous phenomenon rather than a unitary psychological construct (Bőthe, Tóth-Király, Bella, et al., 2021; Grubbs & Perry, 2019).

At the same time, these conclusions should be interpreted within the methodological boundaries of the available evidence. Because the overwhelming majority of included studies employed cross-sectional correlational designs, the present meta-analysis was designed to characterize patterns of association rather than to identify causal mechanisms or developmental pathways. Consequently, observed relationships should be interpreted as descriptive summaries of the existing literature and should not be construed as evidence regarding temporal precedence or etiological processes (Borenstein et al., 2009; Maxwell & Cole, 2007).

### 4.2 Patterns of psychological correlates

Across the examined psychological variables, pornography craving and sexual shame demonstrated the strongest pooled associations with PPU, whereas variables such as extraversion and family environment showed comparatively weak or negligible associations. The relatively strong association with craving is broadly consistent with theoretical accounts emphasizing motivational salience, cue-reactivity, reinforcement learning, and impaired self-regulatory processes in problematic online behaviors (Antons & Brand, 2018; Brand et al., 2019; Robinson & Berridge, 2008). Conversely, the substantial association observed for sexual shame is consistent with literature highlighting the importance of evaluative distress, self-conscious emotions, and negative self-appraisal in individuals who perceive their pornography use as problematic (Grubbs & Perry, 2019; Perry & Whitehead, 2019).

Rather than indicating mutually exclusive explanatory mechanisms, these findings suggest that behavioral dysregulation-related variables and distress-related variables both contribute to the correlational landscape of PPU. The present findings therefore support an integrative interpretation in which multiple psychological processes may be associated with reports of problematic pornography use, although the relative strength of these associations appears to vary across studies and measurement approaches.

Moderate associations were also observed for impulsivity, emotion regulation difficulties, and related self-regulatory constructs. These findings align with broader literature implicating deficits in inhibitory control, emotion regulation, and executive functioning across a range of addictive and compulsive behaviors (Bari & Robbins, 2013; Berking & Wupperman, 2012). Likewise, smaller but generally consistent associations with depressive symptoms, stress, loneliness, and attachment-related variables suggest that PPU frequently co-occurs with broader affective and interpersonal vulnerabilities.

Nevertheless, caution is warranted when interpreting the relative magnitude of these pooled effects. Prediction intervals were frequently wide and, for several correlates, included values approaching zero, indicating that true associations may vary substantially across future studies conducted in different populations and contexts (IntHout et al., 2016; Riley et al., 2011). Accordingly, the pooled estimates reported in the present meta-analysis should be interpreted as average tendencies across heterogeneous studies rather than as universally applicable effect sizes.

This variability is particularly important because wide prediction intervals indicate that statistically significant average effects may not necessarily generalize to every population, setting, or measurement context. Consequently, the present findings emphasize both the consistency of certain broad correlational patterns and the considerable uncertainty surrounding their expected magnitude across individual studies.

Finally, because the available evidence is predominantly cross-sectional, the present findings should not be interpreted as indicating directional, developmental, or causal relationships among the examined psychological variables. Rather, they provide a descriptive synthesis of correlational patterns that may inform future longitudinal, experimental, and mechanistic research.

### 4.3 Measurement-related variability

A central contribution of the present meta-analysis is the explicit examination of measurement heterogeneity as a potential source of variability in reported psychological correlates of problematic pornography use. As discussed in the Introduction, instruments commonly used to assess PPU differ considerably in their conceptual emphasis. Some primarily assess behavioral dysregulation, including impaired control, compulsive engagement, craving, and functional impairment, whereas others place greater emphasis on subjective distress, including guilt, shame, perceived addiction, and moral incongruence (Bőthe et al., 2020; Grubbs et al., 2010; Grubbs & Perry, 2019; Kohut et al., 2020).

The present findings suggest that these differences in operationalization may contribute to variation in the magnitude of observed associations across studies. Descriptively, dysregulation-oriented instruments tended to demonstrate relatively stronger associations with craving, impulsivity, and self-regulatory difficulties, whereas distress-oriented instruments generally showed comparatively stronger associations with shame, guilt, moral incongruence, and religiousness. However, these descriptive patterns should not be interpreted as evidence that these constructs represent distinct latent forms of PPU. Rather, they indicate that observed correlational profiles may differ depending on the conceptual content emphasized by the measurement instrument.

Importantly, substantial conceptual overlap was evident across many commonly used instruments. Several measures include items reflecting both behavioral dysregulation and subjective distress, making any categorical distinction necessarily approximate. Accordingly, the present classification of instruments was intended as a heuristic framework for interpreting variability across studies rather than as a definitive taxonomy of PPU assessment tools. This interpretation is consistent with previous methodological critiques arguing that many existing instruments assess partially overlapping psychological experiences despite using different theoretical terminology (Kohut et al., 2020).

The subgroup analyses further support this cautious interpretation. Although several subgroup differences reached nominal statistical significance, these effects were not observed consistently across psychological correlate domains and generally did not remain robust after correction for multiple comparisons. Consequently, the available evidence does not support strong conclusions regarding systematic differences between dysregulation-oriented and distress-oriented operationalizations.

Instead, the findings suggest that measurement emphasis may account for some—but not all—of the variability observed across studies. Differences in correlational patterns therefore appear more parsimoniously attributable to variation in instrument content than to the existence of clearly separable psychological subtypes. This interpretation is consistent with recent discussions emphasizing that apparent disagreements within the PPU literature may partly arise from differences in operational definitions and measurement strategies rather than fundamentally incompatible conceptualizations (Kohut et al., 2020).

Overall, the present findings underscore the importance of considering **c**onstruct validity and measurement characteristics when interpreting both individual studies and quantitative syntheses of PPU. Future research would benefit from greater conceptual precision, clearer justification for instrument selection, and more explicit consideration of which dimensions of problematic pornography use are being assessed. Such efforts may improve comparability across studies and facilitate more cumulative progress in understanding the psychological correlates of PPU.

### 4.4 Heterogeneity and contextual influences

Substantial between-study heterogeneity was observed across most analyses, with I^2^ values frequently exceeding conventional thresholds for high inconsistency. However, as emphasized in methodological literature, I^2^ should not be interpreted as a direct indicator of the magnitude or practical importance of heterogeneity, because it reflects the proportion of observed variance attributable to between-study differences rather than the absolute dispersion of true effects (Borenstein et al., 2017; Higgins & Thompson, 2002).

For this reason, heterogeneity was interpreted alongside prediction intervals, which provide information regarding the range of effect sizes that might reasonably be expected in future studies conducted under similar assumptions (IntHout et al., 2016; Riley et al., 2011). Across numerous psychological correlates, prediction intervals were considerably wider than confidence intervals and frequently included substantially attenuated effects or values approaching zero. These findings indicate that the strength of associations between psychological variables and PPU is unlikely to be constant across populations, cultural contexts, or methodological conditions.

The moderator analyses provided only limited evidence that broad study-level characteristics systematically accounted for this variability. Continuous moderators, including publication year, sample size, participant age, proportion of female participants, and reliability coefficients, generally demonstrated small and inconsistent effects. Likewise, categorical moderators such as country and timing relative to the COVID-19 pandemic explained relatively little of the observed heterogeneity.

Although several moderator effects reached nominal statistical significance in individual analyses, the overall pattern was inconsistent across psychological correlate domains. Consequently, these findings should be regarded as hypothesis-generating rather than confirmatory and should not be interpreted as evidence for systematic measurement-based subtypes or causal pathways.

Taken together, these findings suggest that the substantial heterogeneity observed throughout the present meta-analysis likely reflects multiple interacting methodological, conceptual, and contextual influences rather than any single identifiable source. Potential contributors include differences in sampling procedures, cultural norms surrounding pornography use, recruitment methods, participant characteristics, instrument selection, and conceptual definitions of PPU.

Rather than viewing this heterogeneity solely as a statistical limitation, it may also be interpreted as reflecting the complexity of the phenomenon under investigation. The variability observed across studies suggests that associations between psychological variables and PPU are context-dependent and may differ according to both methodological and sociocultural conditions. Consequently, the pooled estimates reported in the present study are best understood as summaries of average associations across diverse research contexts rather than universally applicable parameters.

### 4.5 Interpretation in relation to theoretical frameworks

The present findings also have implications for the interpretation of contemporary theoretical models of problematic pornography use. Importantly, however, the current meta-analysis was not designed to evaluate, compare, or validate competing theoretical frameworks. Rather, theoretical models were used as conceptual reference points for interpreting observed correlational patterns within an explicitly measurement-informed framework.

Among the most influential conceptual models, the Interaction of Person–Affect–Cognition–Execution (I-PACE) model proposes that problematic online behaviors emerge through dynamic interactions among individual predispositions, affective responses, cognitive processes, reinforcement learning, and executive functioning (Brand et al., 2016, 2019). Although this framework was developed for addictive online behaviors more broadly rather than specifically for PPU, it has frequently been applied to explain associations involving craving, impaired inhibitory control, and emotion regulation difficulties.

By contrast, the Pornography Problems due to Moral Incongruence (PPMI) model emphasizes that subjective experiences of problematic pornography use may arise when pornography consumption conflicts with an individual's moral, religious, or personal values, even in the absence of pronounced behavioral dysregulation (Grubbs & Perry, 2019). Within this perspective, variables such as guilt, shame, and moral incongruence represent important components of self-perceived problematic use.

The descriptive correlational patterns observed in the present review are broadly compatible with elements of both perspectives. Associations involving craving, impulsivity, and self-regulatory difficulties were generally stronger in studies employing dysregulation-oriented instruments, whereas shame, guilt, moral incongruence, and religiousness tended to demonstrate comparatively stronger associations in studies using distress-oriented measures.

However, these observations should not be interpreted as empirical support for distinct etiological pathways or competing explanatory models. The exploratory subgroup analyses were based on differences in measurement operationalization rather than direct tests of theoretical predictions. Consequently, the present findings cannot determine whether different psychological processes represent independent developmental pathways or merely different manifestations of overlapping psychological experiences.

Instead, the current results are more parsimoniously interpreted as indicating that measurement emphasis influences the correlational profiles observed across studies. Instruments emphasizing behavioral dysregulation naturally tend to show stronger associations with self-regulatory constructs, whereas instruments emphasizing subjective distress are more likely to correlate with evaluative and affective variables. This pattern reflects expected consequences of construct operationalization rather than definitive evidence regarding underlying psychological mechanisms.

This interpretation is consistent with recent methodological discussions suggesting that apparent differences between dysregulation-oriented and distress-oriented conceptualizations may partly reflect differences in measurement content rather than fundamentally distinct forms of problematic pornography use (Kohut et al., 2020). Accordingly, the present findings support a cautious and integrative interpretation in which observed variability is understood primarily as reflecting differences in conceptualization and operationalization across studies.

More broadly, these findings highlight the importance of distinguishing measurement models from etiological models. Although theoretical frameworks provide valuable guidance for organizing empirical findings, evidence derived from cross-sectional correlational studies cannot adjudicate between competing developmental accounts. Longitudinal, experimental, and multimethod research will therefore be required to determine whether the psychological processes emphasized by existing theoretical models represent temporally distinct mechanisms or overlapping aspects of a broader multidimensional phenomenon.

### 4.6 Interpreting distress-related correlates

Among the psychological correlates examined, sexual shame, guilt, moral incongruence, and related evaluative processes demonstrated relatively strong associations with PPU, underscoring the importance of subjective distress in many reports of problematic pornography use. These findings are consistent with previous literature suggesting that individuals' evaluations of their pornography use are often shaped by personal values, cultural expectations, and religious beliefs, in addition to behavioral characteristics of pornography consumption itself (Grubbs & Perry, 2019; Perry & Whitehead, 2019).

Nevertheless, the present findings should not be interpreted as indicating that distress-related correlates are equivalent to behavioral dysregulation. Constructs such as shame and moral incongruence primarily reflect individuals' emotional and cognitive evaluations of their behavior rather than objective indicators of impaired behavioral control. Consequently, strong associations between these variables and PPU may partly reflect overlap in conceptual content among self-report measures rather than direct evidence of compulsive behavioral processes.

Conversely, the findings also do not support interpreting distress-related experiences as entirely independent from behavioral self-regulation. Considerable overlap was observed across measurement approaches, and many instruments incorporate elements of both behavioral dysregulation and subjective distress. This overlap suggests that these psychological processes frequently co-occur and may interact in clinically meaningful ways, even if they are conceptually distinguishable.

Accordingly, the present findings are most consistent with a dimensional rather than categorical interpretation of PPU. Individuals reporting problematic pornography use may experience varying combinations of self-regulatory difficulties, negative affect, moral conflict, and interpersonal distress, with the relative prominence of each process differing across persons and contexts. The available evidence does not support reducing PPU to either behavioral dysregulation alone or moral distress alone.

This interpretation also aligns with the substantial heterogeneity observed throughout the present meta-analysis. Rather than representing discrete subtypes, the psychological correlates identified here appear to reflect partially overlapping domains that contribute to diverse presentations of self-reported problematic pornography use. Such variability reinforces the importance of avoiding overly simplified interpretations of PPU and supports approaches that acknowledge the interaction of behavioral, emotional, cognitive, and contextual influences.

Taken together, the present findings highlight the importance of interpreting distress-related correlates within a broader measurement-informed framework. The observed associations likely reflect both genuine psychological relationships and differences in the constructs emphasized by available assessment instruments. Consequently, future research should continue refining the conceptual boundaries between behavioral dysregulation and subjective distress while recognizing that these processes frequently coexist rather than representing mutually exclusive explanations of problematic pornography use.

### 4.7 Clinical and applied implications

The present findings have several implications for both clinical assessment and future research. First, the results suggest that individuals reporting problematic pornography use may present with a diverse constellation of psychological characteristics rather than a single, uniform clinical profile. Across studies, PPU was associated with variables related to behavioral self-regulation (e.g., craving, impulsivity, emotion regulation difficulties), affective functioning (e.g., depression, anxiety, stress), and evaluative distress (e.g., shame and moral incongruence). This pattern highlights the importance of comprehensive psychological assessment rather than assuming a single underlying mechanism for all individuals reporting problematic pornography use.

From a clinical perspective, the findings support an individualized, formulation-based approach to assessment. Rather than relying solely on total scores from PPU questionnaires, clinicians may benefit from considering which psychological domains are most prominent for a given individual and whether reported difficulties primarily reflect behavioral dysregulation, subjective distress, or a combination of both. Such an approach is consistent with broader principles of evidence-based psychological assessment emphasizing the integration of symptom presentation, contextual factors, and functional impairment.

Importantly, the present findings should not be interpreted as supporting distinct clinical subtypes of PPU. Although descriptive differences were observed across measurement approaches, these differences were generally inconsistent across constructs and frequently accompanied by substantial overlap between instruments. Accordingly, the current evidence does not justify classifying individuals into discrete dysregulation-based versus distress-based categories.

Instead, the results suggest that assessment should distinguish between the specific psychological processes captured by different instruments. Some questionnaires place greater emphasis on impaired behavioral control, whereas others primarily assess subjective distress or perceived problematic use. Awareness of these differences may facilitate more accurate interpretation of assessment results in both clinical and research settings and may reduce the risk of overgeneralizing findings across instruments that operationalize PPU differently.

The present findings also have implications for intervention research. Because the available evidence is correlational, the current meta-analysis cannot determine whether the identified psychological correlates represent causal treatment targets. Consequently, the observed associations should not be interpreted as demonstrating that modifying any particular correlate will necessarily reduce problematic pornography use. Instead, the findings identify psychological domains that consistently co-occur with PPU and therefore warrant further investigation in longitudinal and intervention studies.

More broadly, the results emphasize the importance of conceptual clarity in future clinical research. Greater consistency in the operationalization of PPU, clearer reporting of instrument characteristics, and more precise distinction between behavioral dysregulation and evaluative distress may improve the comparability of intervention studies and facilitate cumulative scientific progress.

### 4.8 Limitations

Several limitations should be considered when interpreting the present findings.First, the available evidence consisted predominantly of cross-sectional studies, precluding conclusions regarding causality or temporal ordering among the examined variables (Maxwell & Cole, 2007). Although some longitudinal evidence has begun to emerge, particularly regarding the prospective role of negative affect (Demirgül et al., 2026), the present meta-analysis was intentionally designed to synthesize correlational evidence rather than evaluate developmental or causal processes.

Second, most primary studies relied exclusively on self-report measures, introducing potential sources of measurement error, shared method variance, recall bias, and social desirability effects. These limitations may have inflated some observed associations, particularly when both PPU and psychological correlates were assessed using self-report questionnaires administered within the same study.

Third, substantial heterogeneity remained across analyses despite the use of random-effects models, prediction intervals, subgroup analyses, and moderator analyses. Although prediction intervals provided a more informative representation of between-study variability than confidence intervals alone, considerable unexplained heterogeneity persisted. This finding suggests that additional methodological and contextual factors—including cultural norms, recruitment strategies, sampling procedures, and differences in instrument content—may contribute to variation in reported effect sizes.

Fourth, the operationalization of PPU varied considerably across studies. Although the present review explicitly addressed this issue by classifying instruments according to their dominant conceptual emphasis, this classification necessarily involved some degree of simplification because many instruments assess overlapping aspects of behavioral dysregulation and subjective distress. Consequently, the measurement categories employed in the present study should be viewed as heuristic rather than definitive.

Fifth, moderator and interaction analyses should be interpreted cautiously. Although several moderator effects reached nominal statistical significance, these findings were generally inconsistent across constructs and were based on relatively small numbers of studies within several subgroups. Accordingly, these analyses were exploratory and hypothesis-generating rather than confirmatory.

Finally, the present meta-analysis was not designed to evaluate competing theoretical models or distinguish among alternative etiological explanations of PPU. The descriptive differences observed across measurement approaches should therefore not be interpreted as evidence supporting distinct developmental pathways or latent subtypes. Instead, they are more appropriately understood as reflecting variation in operationalization and measurement across the existing literature.

Taken together, these limitations underscore the need for future longitudinal, multimethod, and cross-cultural research employing conceptually well-defined assessment instruments and standardized reporting practices. Such work will be essential for clarifying the temporal relationships among psychological correlates, improving construct validity, and determining the extent to which observed variability reflects measurement characteristics versus underlying psychological processes.

## 5 Conclusion

The present meta-analysis provides a comprehensive synthesis of the psychological correlates of problematic pornography use (PPU) while explicitly considering differences in how the construct has been conceptualized and measured across studies. Across the available evidence, PPU was associated with a broad range of psychological variables spanning behavioral self-regulation, emotional functioning, interpersonal processes, and evaluative distress. However, the magnitude of these associations varied considerably across psychological domains and across studies, indicating that PPU cannot be adequately characterized as a single, homogeneous construct.

A central contribution of the present review is its emphasis on measurement heterogeneity as an important consideration when interpreting the literature on PPU. The findings suggest that differences in observed correlational patterns may partly reflect differences in the conceptual emphasis of assessment instruments rather than uniform relationships among psychological constructs. Instruments emphasizing behavioral dysregulation tended to show stronger associations with variables such as craving and impulsivity, whereas instruments emphasizing subjective distress were more strongly associated with shame, guilt, and moral incongruence. Importantly, these descriptive differences were interpreted as reflecting variation in measurement emphasis rather than evidence for distinct psychological subtypes or etiological pathways.

The findings also highlight the importance of interpreting pooled meta-analytic estimates within the context of substantial between-study heterogeneity. Wide prediction intervals and generally high levels of residual heterogeneity indicate that the strength of associations between PPU and psychological variables is likely to vary across populations, cultural contexts, and measurement approaches. Consequently, the pooled effect sizes reported in the present review should be understood as average tendencies across heterogeneous studies rather than universally applicable estimates.

Rather than supporting a single explanatory framework, the present findings underscore the complexity and multidimensional nature of problematic pornography use. The available evidence suggests that behavioral self-regulation, emotional functioning, and evaluative distress are all relevant to understanding PPU, although their relative importance may differ across individuals, contexts, and methods of assessment. Accordingly, future research should continue moving toward greater conceptual precision by clearly distinguishing the constructs being measured and by explicitly justifying the selection of assessment instruments.

Because the present synthesis was based primarily on cross-sectional correlational studies, the findings should not be interpreted as providing evidence regarding causal mechanisms or developmentalpathways. Future research would benefit from longitudinal, multimethod, and theory-informed designs capable of disentangling temporal relationships among psychological variables while minimizing the influence of shared method variance and measurement overlap. Greater use of standardized reporting practices, validated assessment instruments, and cross-cultural sampling may further improve the comparability and cumulative value of future investigations.

Overall, the present meta-analysis suggests that understanding PPU requires a measurement-informed and context-sensitive perspective. The findings indicate that variability in reported psychological correlates reflects, at least in part, differences in operationalization and assessment across studies. Recognizing and explicitly addressing this measurement heterogeneity may contribute to greater conceptual clarity, more accurate interpretation of empirical findings, and stronger foundations for future research on problematic pornography use.

## CRediT authorship contribution statement

**Sonay Sheikhi:** Writing – review & editing, Writing – original draft, Software, Project administration, Methodology, Investigation, Formal analysis, Data curation, Conceptualization. **Shahram Mohammadkhani:** Writing – review & editing, Validation, Supervision, Methodology, Conceptualization. **Mehdi Akbari:** Writing – review & editing, Writing – original draft, Supervision, Methodology, Conceptualization.

## Ethical approval

Not applicable (systematic review and meta-analysis).

## Funding

No external funding was received for this research.

## Declaration of competing interest

The authors declare that they have no known competing financial interests or personal relationships that could have appeared to influence the work reported in this paper.

## Data Availability

Data will be made available on request.

## Appendix A Supplementary data

Table S1. Characteristics of studies included in the meta-analysis.Study characteristics, including publication year, country, COVID-19 pandemic period, sample size, percentage of female participants, mean age, psychological factors assessed, measurement instruments, PPU assessment instrument, and classification of the PPU instrument as distress-oriented or dysregulation-oriented. Abbreviations for psychological factors and measurement instruments are defined in the accompanying notes. Table S2. Summary of study quality assessment.Quality assessment of the included studies using a modified Kmet et al. (2004) checklist. Studies were evaluated across domains including study objectives and design, participant selection and characteristics, intervention and blinding procedures where applicable, outcome measurement, sample size, analytical methods, variance reporting, confounding control, reporting quality, and whether conclusions were supported by the results. Ratings were categorized as Yes, Partial, No, or Not Applicable. Inter-rater agreement was substantial (Cohen's κ = 0.82).
