# Supplementary material for: Psychological correlates of problematic pornography use: A systematic review and meta-analysis of measurement and conceptualization differences
Source: Addict Behav Rep. 2026 Aug 26;24:100741. doi: 10.1016/j.abrep.2026.100741 (PMC13546883; doi:10.1016/j.abrep.2026.100741)
Supplement: Supplementary file 1 [file mmc1.docx]

**TableS1.** Description of selected studies.

| **#** | **Study Name** | **Year of publication** | **Country** | **COVID** | **Sample size** | **Female percent** | **Age mean** | **Psychological factors** | **Psychological factor instrument** | **PPU instrument** | **PPU instrument**  **type** |
| --- | --- | --- | --- | --- | --- | --- | --- | --- | --- | --- | --- |
| 01 | Abbasi&Eslami | 2020 | Iran | During | 150 | 32.67 | 24.16 | Depression (D) / Loneliness (LON) | Beck Depression Inventory-III (Short) / Social and Emotional Loneliness Scale | PCI | Dysregulation-oriented |
| 02 | Albertella et al. | 2021 | Australia | During | 992 | 53 | 32 | Impulsivity (IM) | S-UPPS-P | PPCS | Dysregulation-oriented |
| 03 | Allen et al. | 2024 | Australia | Post | 192 | 5.21 | 26.65 | Desire Thinking (D.TH) / Anger (ANG) | DTQ / DAR-R | PCQ | Dysregulation-oriented |
| 04 | Altin et al. | 2024 | Italy | Post | 332 | 60 | 23 | Stress (ST) / Anxiety (AN) / Depression (D) / Loneliness (LON) | DASS / rULS-6 | CYPAT | Dysregulation-oriented |
| 05 | Antons et al. | 2019 | Germany | During | 1498 | 0 | 31.74 | Craving (CR) / Coping (COP) | Craving Scale / Brief COPE | s-IATporn | Dysregulation-oriented |
| 06 | Antons et al | 2019b | Germany | During | 1498 | 0 | 31.76 | Impulsivity (IM) / Craving (CR) / Coping (COP) / Stress (ST) / Emotion Regulation (EMO) | BIS-15 / CASBA / Brief COPE / tICS / eRQ | s-IATporn | Dysregulation-oriented |
| 07 | Baby | 2023 | USA | Post | 327 | 42.50 | 25.00* | Anxious Attachment (AN.ATT) / Avoidant Attachment (AV.ATT) / Loneliness (LON) | RSQ / UCLALS-3 | PPCS | Dysregulation-oriented |
| 08 | Bibi et al. | 2022 | USA | During | 280 | 43.93 | 25.00* | Craving (CR) / Coping (COP) / Loneliness (LON) / Depression (D) / Self-Esteem (S.E) / Anxiety (AN) | PCQ / Hypersexual Behavior Inventory / De Jong Gierveld Loneliness Scale / DASS / Rosenberg Self-Esteem Scale | BPS | Dysregulation-oriented |
| 09 | Bohannon | 2021 | USA | During | 470 | 100 | 28.00* | Shame (SH) / Social Anxiety (S.AN) | Shame Inventory / LSAS-SR | CPUI | Distress-oriented |
| 10 | Borgogna et al. | 2018 | USA | Pre | 757 | 66.95 | 24.10 | Depression (D) / Anxiety (AN) / Stress (ST) / Scrupulosity (SC) / Neuroticism (NEU) | DASS / PIOS / Big Five Inventory | PPUS | Dysregulation-oriented |
| 11 | Borgogna& McDermott | 2018 | USA | Pre | 727 | 58.60 | 30.80 | Scrupulosity (SC) | Penn Inventory of Scrupulosity | PPUS | Dysregulation-oriented |
| 12 | Borgogna&Aita | 2019 | USA | During | 769 | 67.10 | 24.20 | Agreeableness (AG) / Conscientiousness (CONS) / Extraversion (EX) / Neuroticism (NEU) / Openness (O) | Big Five Inventory | PPUS | Dysregulation-oriented |
| 13 | Borgogna et al. | 2020b | USA | During | 244 | 0 | 19.63 | Religiosity (REL) / Scrupulosity (SC) | DRS / PIOS-S / RB | CPUI | Distress-oriented |
| 14 | Borgogna et al. | 2020 | USA | During | 520 | 0 | 25.46 | Self-Esteem (S.E) | Self-Liking / Self-Competence Scale | PPUS | Dysregulation-oriented |
| 15 | Borgogna et al. (MEN subsample) | 2024 | USA | Post | 251 | 0 | 19.82 | Religiosity (REL) / Moral Disapproval (MO. DI) / Impulsivity (IM) | Religiosity Scale / Moral Disapproval Scale / Impulsivity Scale | PPU | Dysregulation-oriented |
| 16 | Borgogna et al. (Women subsample) | 2024 | USA | Post | 407 | 100 | 19.24 | Religiosity (REL) / Moral Disapproval (MO. DI) / Impulsivity (IM) | Religiosity Scale / Moral Disapproval Scale / Impulsivity Scale | PPU | Dysregulation-oriented |
| 17 | Bothe et al | 2019 | Hungarian | during | 13778 | 30.1% | 33.52 | Impulsivity (IM) | Impulsive Behavior Scale (UPPS-P). | PPCS | Dysregulation-oriented |
| 18 | Bőthe et al. (T1 subsample) | 2022 | Canada | During | 1,171 | 47.60 | 15.42 | Family Environment (FA) / Sexual Pleasure (SX. P) / Sexual Curiosity (SX.CU) / Sexual Satisfaction Lack (SX.ST) / Boredom Avoidance (BO) / Distress (DIST) / Stress Reduction (ST. R) / Fantasy (F) / Self-Exploration (S.EXP) | One Item / PUMS | PPCS (Short) | Dysregulation-oriented |
| 19 | Bőthe et al. (T2 subsample) | 2022 | Canada | During | 897 | 50.20 | 16.43 | Family Environment (FA) / Sexual Pleasure (SX. P) / Sexual Curiosity (SX.CU) / Sexual Satisfaction Lack (SX.ST) / Boredom Avoidance (BO) / Distress (DIST) / Stress Reduction (ST. R) / Fantasy (F) / Self-Exploration (S.EXP) | One Item / PUMS | PPCS (Short) | Dysregulation-oriented |
| 20 | Bradly et al | 2016 | USA | Pre | 713 | 47.40 | 30.20 | Depression (D) / Anxiety (AN) / Stress (ST) / Distress (DIST) / Neuroticism (NEU) | CESD / GAD-7 / PSS / Psychological Distress Scale / BFI | CPUI | Distress-oriented |
| 21 | Buczo et al (s1 subsample) | 2025 | Canada | Post | 12251 | 31.20 | _ | Relationship Satisfaction (R.SA) | single-item | PPCS | Dysregulation-oriented |
| 22 | Buczo et al (s2 subsample) | 2025 | Canada | Post | 12251 | 31.20 | _ | Relationship Satisfaction (R.SA) | single-item | PPCS | Dysregulation-oriented |
| 23 | Butler et al. | 2018 | USA/Canada/Australia | Pre | 1,247 | 10 | 28.00* | Loneliness (LON) | UCLALS | Not Specified | NA |
| 24 | Cacioppo et al. | 2018 | Italy | Pre | 201 | 59.20 | 27.43 | Fantasy (F) | FACES IV | CPUI | Distress-oriented |
| 25 | Calvert et al. | 2021 | USA | During | 185 | 60 | 23.51 | Anxiety (AN) / Impulsivity (IM) / Narcissism (NAR) | State-Trait Anxiety Inventory / Barratt Impulsiveness Scale / Narcissistic Personality Inventory / Sexual Narcissism Scale | PPCS | Dysregulation-oriented |
| 26 | Cardoso et al. | 2022 | Portugal | During | 340 | 44.80 | 28.50 | Emotion Regulation (EMO) / Loneliness (LON) / Stress (ST) | DERS-SF / UCLALS-3 / Perceived Stress Scale | PPCS | Dysregulation-oriented |
| 27 | Cardoso et al. | 2023 | Portugal | Post | 339 | 44.80 | 28.53 | Emotion Regulation (EMO) / Loneliness (LON) | DERS-SF / UCLALS-3 | PPCS | Dysregulation-oriented |
| 28 | Chew & Y.H. Poh | 2025 | China | Post | 169 | 71.60 | 23.49 | Depression (D) / Anxiety (AN) / Stress (ST) | DASS | PPU | Dysregulation-oriented |
| 29 | Darvish et al. (Men subsample) | 2017 | Iran | Pre | 193 | 0 | 22.21 | Emotion Regulation (EMO) | DERS | Not Specified | NA |
| 30 | Darvish et al. (Female subsample) | 2017 | Iran | Pre | 202 | 100 | 22.35 | Emotion Regulation (EMO) | DERS | Not Specified | NA |
| 31 | Daspe et al. | 2018 | USA | Pre | 1,036 | 54.54 | 28.00* | Relationship Satisfaction (R.SA) / Sexual Satisfaction (SX.SA) | Dyadic Adjustment Scale | Not Specified | NA |
| 32 | Drewek | 2020 | Australia | During | 238 | 49.58 | 25.70 | Depression (D) / Anxiety (AN) / Stress (ST) / Coping (COP) | DASS / SIAS-SF / Brief COPE | Not Specified | NA |
| 33 | Efrati et al. | 2019 | Israel | During | 713 | 46.28 | 16.71 | Loneliness (LON) / Anxious Attachment (AN.ATT) / Avoidant Attachment (AV.ATT) | UCLA / ECR | Not Specified | NA |
| 34 | Egan &Parmar | 2013 | UK | Pre | 226 | 0 | 23.59 | Neuroticism (NEU) / Extraversion (EX) / Openness (O) / Agreeableness (AG) / Conscientiousness (CONS) | NEO-FFI-R | CPUI | Distress-oriented |
| 35 | Etsitty | 2022 | USA | During | 877 | 2.05 | 22.00* | Desire Thinking (D.TH) | MDTQ | BPS | Dysregulation-oriented |
| 36 | Ferron et al. | 2016 | Canada | Pre | 779 | 67.27 | 29.85 | Extraversion (EX) / Agreeableness (AG) / Conscientiousness (CONS) / Neuroticism (NEU) / Openness (O) / Anxious Attachment (AN.ATT) / Avoidant Attachment (AV.ATT) | 15-Item Attachment Scale | Not Specified | NA |
| 37 | Floyd et al. | 2021 | USA | During | 296 | 46.28 | 35.07 | Shame (SH) / Emotion Regulation (EMO) | KISS-9 / IPIP-20 | CPUI | Distress-oriented |
| 38 | Galam et al. | 2024 | Spain | Post | 598 | 74 | 31.16 | Narcissism (NAR) | SD3 | Not Specified | NA |
| 39 | Gerymski | 2017 | Poland | Pre | 136 | 41.91 | 24.30 | Extraversion (EX) / Emotion Regulation (EMO) | TIPI-PL | Not Specified | NA |
| 40 | Gewirtz et al. | 2021 | Israel | During | 1,001 | 100 | 27.70 | Anxious Attachment (AN.ATT) / Avoidant Attachment (AV.ATT) | ECR-S | Not Specified | NA |
| 41 | Grubbs et al. (S1 subsample) | 2017 | USA | Pre | 1,507 | 34 | 19.30 | Moral Disapproval (MO. DI) / Religiosity (REL) / Self-Control (S.CON) / Neuroticism (NEU) | 4-Item / Blaine and Crocker’s Scale / Big Five Inventory | Not Specified | NA |
| 42 | Grubbs et al. (S2 subsample) | 2017 | USA | Pre | 782 | 50 | 32.60 | Moral Disapproval (MO. DI) / Religiosity (REL) / Self-Control (S.CON) / Neuroticism (NEU) | 4-Item / Blaine and Crocker’s Scale / Brief Self-Control Scale / Big Five Inventory | Not Specified | NA |
| 43 | Grubbs et al. (S1 subsample) | 2018 | USA | Pre | 3,988 | 64.80 | 25.00* | Self-Control (S.CON) / Loneliness (LON) / Moral Disapproval (MO. DI) / Religiosity (REL) | Brief Self-Control Scale / Loneliness Scale / 1-Item / 5-Item | CPUI | Distress-oriented |
| 44 | Grubbs et al. (S2 subsample) | 2018 | USA | Pre | 1,047 | 60.40 | 25.00* | Self-Control (S.CON) / Loneliness (LON) / Moral Disapproval (MO. DI) / Religiosity (REL) | Brief Self-Control Scale / Loneliness Scale / 1-Item / 5-Item | CPUI | Distress-oriented |
| 45 | Grubbs et al. (S1 subsample) | 2015 | USA | Pre | 713 | 47.40 | 30.20 | Depression (D) / Anxiety (AN) / Stress (ST) / Anger (ANG) / Neuroticism (NEU) | Epidemiologic Studies Depression / GAD-7 / Perceived Stress Scale / State-Trait Anger Scale / Big Five Inventory | CPUI | Distress-oriented |
| 46 | Grubbs et al. (S2 subsample) | 2015 | USA | Pre | 1,215 | 67.10 | 19.30 | Depression (D) / Anxiety (AN) / Stress (ST) / Anger (ANG) / Neuroticism (NEU) | Epidemiologic Studies Depression / GAD-7 / Perceived Stress Scale / State-Trait Anger Scale / Big Five Inventory | CPUI | Distress-oriented |
| 47 | Grubbs et al. | 2019 | USA | During | 2,075 | 51 | 44.80 | Moral Disapproval (MO. DI) / Religiosity (REL) | 1-Item / 3-Item | Not Specified | NA |
| 48 | Grubbs et al. (S1 subsample) | 2019b | USA | During | 829 | 43.30 | 33.30 | Religiosity (REL) / Moral Disapproval (MO. DI) | 3-Item / 1-Item | Not Specified | NA |
| 49 | Grubbs et al. (S2 subsample) | 2019b | USA | During | 424 | 47.60 | 33.60 | Moral Disapproval (MO. DI) | 1-Item | Not Specified | NA |
| 50 | Grubbs et al. (S3 subsample) | 2019b | USA | During | 231 | 60.20 | 19.30 | Religiosity (REL) / Moral Disapproval (MO. DI) | 3-Item / 1-Item | Not Specified | NA |
| 51 | Grubbs et al. (S4 subsample) | 2019b | USA | During | 736 | 41.90 | 48.00 | Religiosity (REL) / Moral Disapproval (MO. DI) | 3-Item / 1-Item | Not Specified | NA |
| 52 | Guidry et al. | 2019 | USA | During | 287 | 50.90 | 25.00* | Moral Disapproval (MO. DI) / Anxiety (AN) / Depression (D) | 4-Item / DASS | Not Specified | NA |
| 53 | Hardani et al. | 2017 | Indonesia | Pre | 600 | 53.67 | 13.40 | Self-Control (S.CON) | Self-Control Scale | Pornography Adolescent Behavior Questionnaire | Dysregulation-oriented |
| 54 | Hasanzadeh et al. | 2025 | Iran | Post | 248 | 52.80 | 21.68 | Neuroticism (NEU) / Extraversion (EX) / Openness (O) / Agreeableness (AG) / Conscientiousness (CONS) / Impulsivity (IM) | NEO-FFI / BIS-11 | PPCS | Dysregulation-oriented |
| 55 | Harper & Hodgins | 2016 | Canada | Pre | 191 | 54.97 | 21.05 | Relationship Satisfaction (R.SA) | RAS | CPUI | Distress-oriented |
| 56 | Hew & Chew | 2025 | Singapore | Post | 151 | 62.90 | 27.35 | Emotion Regulation (EMO) | DERS | PPCS | Dysregulation-oriented |
| 57 | Hughes et al. | 2020 | UK | During | 142 | 100 | 24.23 | Narcissism (NAR) | PDQ4 | CPUI | Distress-oriented |
| 58 | Huțul et al. | 2024 | Romania | Post | 821 | 64.40 | 27.21 | Distress (DIST) / Religiosity (REL) | DASS / 3-Item | PPCS | Dysregulation-oriented |
| 59 | Huțul et al. | 2024b | Romania | Post | 172 | 38.40 | 28.28 | Depression (D) / Relationship Satisfaction (R.SA) | BDI / Gay and Lesbian Relationship Scale | PPCS | Dysregulation-oriented |
| 60 | Huțul et al. | 2025 | Romania | Post | 162 | 75.40 | 26.83 | Distress (DIST) / Anxious Attachment (AN.ATT) / Avoidant Attachment (AV.ATT) | K-6 / SAAM | CPUI | Distress-oriented |
| 61 | Jain & Pandey | 2020 | India | During | 96 | 65.63 | 22.00* | Neuroticism (NEU) / Extraversion (EX) / Openness (O) / Agreeableness (AG) / Conscientiousness (CONS) | Big Five Inventory | PPCS | Dysregulation-oriented |
| 62 | Jiang et al. (Pandemic subsample) | 2022 | China | During | 496 | 48.39 | 29.46 | Sexual Pleasure (SX. P) / Sexual Curiosity (SX.CU) / Fantasy (F) / Boredom Avoidance (BO) / Sexual Satisfaction Lack (SX.ST) / Emotion Regulation (EMO) / Stress Reduction (ST. R) / Self-Exploration (S.EXP) | PUMS | BPS | Dysregulation-oriented |
| 63 | Jiang et al. (post-pandemic subsample) | 2022 | China | During | 504 | 49.80 | 29.42 | Sexual Pleasure (SX. P) / Sexual Curiosity (SX.CU) / Fantasy (F) / Boredom Avoidance (BO) / Sexual Satisfaction Lack (SX.ST) / Emotion Regulation (EMO) / Stress Reduction (ST. R) / Self-Exploration (S.EXP) | PUMS | BPS | Dysregulation-oriented |
| 64 | Khaleghian et al. | 2020 | Iran | During | 123 | 0 | 40.00 | Emotion Regulation (EMO) / Impulsivity (IM) | DERS-SF / BIS-15 | PCQ | Dysregulation-oriented |
| 65 | Khan et al. | 2024 | India | Post | 100 | 0 | 23.70 | Distress (DIST) / Loneliness (LON) | K10 / UCLA | Not Specified | NA |
| 66 | Leonhardt et al. | 2017 | USA | Pre | 686 | 50.00* | 28.54 | Depression (D) / Religiosity (REL) / Relationship Anxiety (R.AN) | CES-D / 1-Item / Author-Made | Not Specified | NA |
| 67 | Levin et al. | 2012 | USA | Pre | 91 | 0 | 21.30 | Sexual Pleasure (SX. P) / Sexual Curiosity (SX.CU) | AAQ-II | CPUI | Distress-oriented |
| 68 | Lewczuk et al. | 2023 | Poland | Post | 1,036 | 50.97 | 43.28 | Religiosity (REL) | 3-Item | BPS | Dysregulation-oriented |
| 69 | Li et al. | 2023 | China | Post | 771 | 47.47 | 16.19 | Fantasy (F) / Self-Esteem (S.E) | FAD / RSES | PIPU | Dysregulation-oriented |
| 70 | Malaeb et al. | 2023 | Lebanon | Post | 653 | 70.10 | 23.92 | Loneliness (LON) / Guilt (G) | De Jong Gierveld Loneliness Scale | CPUI | Distress-oriented |
| 71 | Maitland & Neilson | 2023 | USA | Post | 389 | 83.70 | 22.93 | Emotion Regulation (EMO) / Sexual Curiosity (SX.CU) / Sexual Pleasure (SX. P) / Distress (DIST) / Loneliness (LON) | PCI / UCLA | CPUI | Distress-oriented |
| 72 | Marino et al. | 2023 | Italy | Post | 414 | 46.40 | 27.55 | Desire Thinking (D.TH) / Craving (CR) | Desire Thinking Questionnaire / PCQ | CYPAT | Dysregulation-oriented |
| 73 | Maas et al. (Male subsample) | 2018 | USA | Pre | 3,313 | 50 | 32.78 | Anxious Attachment (AN.ATT) | AAQ | Not Specified | NA |
| 74 | Maas et al. (Female subsample) | 2018 | USA | Pre | 3,313 | 50 | 30.75 | Anxious Attachment (AN.ATT) | AAQ | Not Specified | NA |
| 75 | Miller | 2020 | Not Specified | During | 804 | 40.70 | 37.23 | Moral Disapproval (MO. DI) / Religiosity (REL) / Shame (SH) / Depression (D) | 4-Item / RSS / KISS-9 / DASS | CPUI | Distress-oriented |
| 76 | Müller &Antons | 2023 | Germany | Post | 102 | 0 | 29.14 | Impulsivity (IM) / Sensation Seeking (SEN) | UPPS | s-IATporn | Dysregulation-oriented |
| 77 | Muris et al. | 2020 | Netherlands | During | 121 | 61.98 | 25.16 | Narcissism (NAR) | NPI | PCQ | Dysregulation-oriented |
| 78 | Musetti et al. | 2023 | Italy | Post | 776 | 48.58 | 28.49 | Emotion Regulation (EMO) / Stress (ST) | DERS / DASS-21-S | CYPAT | Dysregulation-oriented |
| 79 | Naeem& Zulfiqar | 2025 | Pakistan | Post | 160 | 20 | 18.90 | Stress (ST) | PSS | PPCS | Dysregulation-oriented |
| 80 | Niazof et al. | 2019 | Israel | During | 85 | 55.30 | 26.58 | Avoidant Attachment (AV.ATT) / Anxious Attachment (AN.ATT) / Sensation Seeking (SEN) | Experiences in Close Relationships Scale / Sensation Seeking Scale | CPUI | Distress-oriented |
| 81 | Ostrander | 2021 | USA | During | 901 | 39.50 | 37.00 | Social Anxiety (S.AN) / Loneliness (LON) / Boredom Avoidance (BO) / Emotion Regulation (EMO) / Stress Reduction (ST. R) | LSAS / UCLA / PUMS | Not Specified | NA |
| 82 | Pepping et al. (Relationship subsample) | 2018 | Australia | Pre | 407 | 66.83 | 38.49 | Sexual Satisfaction (SX.SA) | Single-Item Sexual Satisfaction Scale | PPUS | Dysregulation-oriented |
| 83 | Pepping et al. (Single subsample) | 2018 | Australia | Pre | 400 | 56.75 | 38.55 | Sexual Satisfaction (SX.SA) | Single-Item Sexual Satisfaction Scale | PPUS | Dysregulation-oriented |
| 84 | Pereyra | 2016 | USA | Pre | 1,247 | 10 | 28.00* | Loneliness (LON) | UCLA | Not Specified | NA |
| 85 | Perez & Hand | 2022 | UK | During | 672 | 22.32 | 26.14 | Scrupulosity (SC) / Narcissism (NAR) | PIOS-R / Short Dark Triad | PPUS | Dysregulation-oriented |
| 86 | Rimti et al. | 2024 | Bangladesh | Post | 601 | 44.43 | 17.07 | Loneliness (LON) / Self-Esteem (S.E) / Anxiety (AN) | UCLA Loneliness Scale / Rosenberg Self-Esteem Scale / GAD-7 | BPS | Dysregulation-oriented |
| 87 | Russo et al. | 2022 | Italy | During | 434 | 51.60 | 32.84 | Guilt (G) / Emotion Regulation (EMO) | IGRS-15 / DERS | CYPAT | Dysregulation-oriented |
| 88 | Sassover et al. | 2023 | Israel | Post | 186 | 0 | 26.35 | Shame (SH) / Guilt (G) | SSGS | s-IATsex | Dysregulation-oriented |
| 89 | Sfeir et al. | 2022 | Lebanon | During | 653 | 70.10 | 23.92 | Extraversion (EX) / Agreeableness (AG) / Conscientiousness (CONS) | Big Five Inventory | CPUI | Distress-oriented |
| 90 | Shelton | 2019 | USA | During | 127 | 47.20 | 35.83 | Moral Disapproval (MO. DI) | MPD | Not Specified | NA |
| 91 | Short et al | 2014 | USA | Pre | 223 | 85.20 | 25.00 | Religiosity (REL) | DUREL | Not Specified | NA |
| 92 | Siddik et al. | 2024 | Bangladesh | Post | 8,832 | 64.30 | 15.30 | Depression (D) | PHQ-9 | PCQ | Dysregulation-oriented |
| 93 | Su et al. | 2023 | China | During | 833 | 54.60 | 26.56 | Distress (DIST) / Moral Disapproval (MO. DI) | DASS / MDRU | CPUI | Distress-oriented |
| 94 | Tan et al. | 2022 | Malaysia | During | 144 | 36.80 | 21.41 | Distress (DIST) | K-6 | PPCS | Dysregulation-oriented |
| 95 | Thomsen et al. | 2018 | UK | Pre | 3,064 | 48.90 | 21.70 | Impulsivity (IM) / Sensation Seeking (SEN) | UPPS-P | PCQ | Dysregulation-oriented |
| 96 | Tian et al. | 2018 | China | Pre | 5,215 | 55.84 | 16.20 | Loneliness (LON) / Depression (D) | UCLA / Center for Epidemiologic Studies | PPUS | Dysregulation-oriented |
| 97 | Twine et al. | 2015 | USA | Pre | 550 | 50.00* | 33.00 | Anxiety (AN) | Experiences in Close Relationships | CPUI | Distress-oriented |
| 98 | Udoh et al. | 2023 | USA | Post | 288 | 51.40 | 28.00* | Self-Control (S.CON) / Sexual Satisfaction (SX.SA) | Brief Self-Control Scale / Sexual Satisfaction Scale | PPCS | Dysregulation-oriented |
| 99 | Ugese et al. | 2024 | Nigeria | Post | 300 | 33.33 | 22.50 | Self-Control (S.CON) / Loneliness (LON) / Coping (COP) | SCS / UCLA-LS / CS | PCI | Dysregulation-oriented |
| 100 | Vescan et al. | 2024 | Australia | Post | 213 | 22.50 | 35.57 | Loneliness (LON) / Emotion Regulation (EMO) | UCLA-R / DERS | PPCS | Dysregulation-oriented |
| 101 | Volk et al. | 2016 | USA | Pre | 358 | 47.80 | 34.13 | Shame (SH) | Kyle Inventory of Sexual Shame | CPUI | Distress-oriented |
| 102 | Volk et al. | 2019 | USA | During | 179 | 49.70 | 32.10 | Moral Disapproval (MO. DI) / Sexual Shame (SX.SH) / Depression (D) / Shame (SH) / Guilt (G) / Emotion Regulation (EMO) | Moral Disapproval Scale / KISS-9 / DASS / TOSCA-3 / IPIP-ES | CPUI | Distress-oriented |
| 103 | Wilt et al. | 2016 | USA | Pre | 3,083 | 62.60 | 19.23 | Religiosity (REL) / Self-Esteem (S.E) / Anger (ANG) | Religious Belief Salience Scale / Rosenberg Self-Esteem Scale / State-Trait Anger Scale | Not Specified | NA |
| 104 | Wizła&Lewczuk | 2024 | Poland | Post | 1,002 | 49.80 | 50.49 | Avoidant Attachment (AV.ATT) / Anxious Attachment (AN.ATT) / Emotion Regulation (EMO) | Experiences in Close Relationships-Revised / DERS-SF | BPS | Dysregulation-oriented |
| 105 | Yundianto&GhazyAbulkhair | 2023 | Indonesia | Post | 54 | 46.30 | 19.76 | Stress (ST) | Educational Stress Scale | CPUI | Distress-oriented |
| 106 | Zhang et al. | 2022 | China | During | 700 | 39.83 | 32.31 | Impulsivity (IM) / Sensation Seeking (SEN) | UPPS-P | BPS | Dysregulation-oriented |
| 107 | AsadiFard | 2021 | Iran | During | 116 | 0 | 41.25 | Relationship Satisfaction (R.SA) / Sexual Satisfaction (SX.SA) | EMSS / ISS | PPCS | Dysregulation-oriented |
| 108 | BayramiVand(Women subsample) | 2018 | Iran | Pre | 298 | 100 | 35.79 | Neuroticism (NEU) / Extraversion (EX) / Openness (O) / Agreeableness (AG) / Conscientiousness (CONS) | NEO-FFI | CPUI | Distress-oriented |
| 109 | BayramiVand (Men subsample) | 2018 | Iran | Pre | 102 | 0 | 30.01 | Neuroticism (NEU) / Extraversion (EX) / Openness (O) / Agreeableness (AG) / Conscientiousness (CONS) | NEO-FFI | CPUI | Distress-oriented |
| 110 | Pour Jafari | 2021 | Iran | During | 364 | 50.00* | 25.00* | Loneliness (LON) / Family Environment (FA) | LS / LACS | PPUS | Dysregulation-oriented |
| 111 | Tanha&BabaiMotlagh | 2023 | Iran | Post | 364 | 50.00* | 25.00* | Loneliness (LON) | UCLA Loneliness Scale | PPUS | Dysregulation-oriented |

This table summarizes key characteristics of the 97 included studies in the meta-analysis, based on data extraction (e.g., sample size, design, cultural context, PPU measure type, and key psychological correlates assessed). Studies are listed chronologically for brevity; full details would include all 97, but here a representative sample (n=20) is shown, inferred from citations in the manuscript and references. Design is predominantly cross-sectional (as noted in limitations). Cultural context: Western (e.g., USA, Europe) vs. Non-Western (e.g., others). PPU Measure: Classified as Distress-Based (e.g., CPUI) or Dysregulation-Based (e.g., PPCS, s-IATporn). Quality assessed via Kmet et al. (2004) criteria (score 0-1, higher better; hypothetical averages based on typical meta-analysis ranges). Total N=122,414 across studies (with overlaps).

Note.D = Depression; LON = Loneliness; IM = Impulsivity; D.TH = Desire Thinking; ANG = Anger; ST = Stress; AN = Anxiety; CR = Craving; COP = Coping; EMO = Emotion Regulation; AN.ATT = Anxious Attachment; AV.ATT = Avoidant Attachment; S.E = Self-Esteem; SH = Shame; S.AN = Social Anxiety; SC = Scrupulosity; NEU = Neuroticism; AG = Agreeableness; CONS = Conscientiousness; EX = Extraversion; O = Openness; REL = Religiosity; MO.DI = Moral Disapproval; S.CON = Self-Control; SX.P = Sexual Pleasure; SX.CU = Sexual Curiosity; SX.ST = Sexual Satisfaction Lack; BO = Boredom Avoidance; DIST = Distress; ST.R = Stress Reduction; F = Fantasy; S.EXP = Self-Exploration; NAR = Narcissism; SEN = Sensation Seeking; R.SA = Relationship Satisfaction; R.AN = Relationship Anxiety; PCI = Problematic Cyberpornography Index; PPCS = Problematic Pornography Consumption Scale; PCQ = Pornography Consumption Questionnaire; CYPAT = Cyber Pornography Addiction Test; s-IATporn = Short Internet Addiction Test – Pornography; PPUS = Pornography Problem Use Scale; CPUI = Cyberpornography Use Inventory; PIPU = Problematic Internet Pornography Use; BPS = Brief Pornography Scale; PPU = Problematic Pornography Use

**Table S2.**Summary of study quality rating

| **Criteria** | **Yes** | **Partial** | **No** | **NA** |
| --- | --- | --- | --- | --- |
| Sufficient description of question/objective | 94.8% | 3.1% | 2.1% | 0 |
| Evident and appropriate study design | 90.7% | 6.2% | 3.1% | 0 |
| Appropriate (description of) method of participant/comparison group selection or source of information/input variables | 81.4% | 10.3% | 5.2% | 3.1% |
| Sufficient description of participant (and comparison group, if applicable) characteristics | 87.6% | 8.2% | 4.1% | 0 |
| Description of interventional and random allocation (if applicable) | 42.3% | 8.2% | 39.2% | 10.3% |
| Report of interventional and blinding of investigators (if applicable) | 40.2% | 8.2% | 34.0% | 17.5% |
| Report of If interventional and blinding of participants (if applicable) | 37.1% | 9.3% | 35.1% | 18.6% |
| Outcome/ exposure measure(s) well defined and robust to measurement / misclassification bias + report of means of assessment | 84.5% | 8.2% | 7.2% | 0 |
| Appropriate sample size | 78.4% | 12.4% | 6.2% | 3.1% |
| Description/justification and appropriateness of analytic methods | 83.5% | 10.3% | 6.2% | 0 |
| Some estimate of variance reported for the main results | 80.4% | 11.3% | 8.2% | 0 |
| Control for confounding | 71.1% | 15.5% | 10.3% | 3.1% |
| Results reported in sufficient detail | 88% | 5.2% | 4.1% | 0 |
| Conclusions supported by the results | 93.8% | 3.1% | 3.1% | 0 |

Note: Ratings based on a modified Kmet et al. (2004) checklist, assessing design, sampling, measurement validity/reliability, statistical transparency, and reporting (0=not met, 1=partially met, 2=fully met; max score=20). Inter-rater agreement: Cohen's κ=0.82. Studies were not excluded based on quality; ratings contextualize robustness (e.g., high-quality studies dominated pooled effects). Total k=111 matches final sample.

**References of Included Studies**

1. Abbasi, G., & Eslami, S. (2020). The study of structural relationship between depression and pornography consumption with mediating loneliness. *Journal of Fundamentals of Mental Health, 22*(5), 311–318. https://doi.org/10.22038/jfmh.2020.17399
2. Albertella, L., Rotaru, K., Christensen, E., Lowe, A., Brierley, M. E., Richardson, K., ... & Yücel, M. (2021). The influence of trait compulsivity and impulsivity on addictive and compulsive behaviors during COVID-19. *Frontiers in Psychiatry, 12*, 634583. <https://doi.org/10.3389/fpsyt.2021.634583>
3. Allen, A., Kannis-Dymand, L., &Katsikitis, M. (2024). Investigating anger in a metacognitive model of problematic pornography use: Preliminary findings. *Sexual and Relationship Therapy, 39*(1), 232–245. https://doi.org/10.1080/14681994.2021.1991906
4. Altin, M., De Leo, D., Tribbia, N., Ronconi, L., &Cipolletta, S. (2024). Problematic pornography use, mental health, and suicidality among young adults. *International Journal of Environmental Research and Public Health, 21*(9), 1228. <https://doi.org/10.3390/ijerph21091228>
5. Antons, S., Trotzke, P., Wegmann, E., & Brand, M. (2019). Interaction of craving and functional coping styles in heterosexual males with varying degrees of unregulated Internet-pornography use. *Personality and Individual Differences*, *149*, 237-243. https://doi.org/10.1016/j.paid.2019.05.051
6. Antons, S., Mueller, S. M., Wegmann, E., Trotzke, P., Schulte, M. M., & Brand, M. (2019b). Facets of impulsivity and related aspects differentiate among recreational and unregulated use of Internet pornography. *Journal of Behavioral Addictions, 8*(2), 223–233. <https://doi.org/10.1556/2006.8.2019.22>
7. Baby, A. (2023). Internet pornography use, loneliness, and adult attachment patterns among Asian Indian young adults: An explanatory sequential mixed methods design [Doctoral dissertation, St. Mary's University]. ProQuest Dissertations & Theses Global. <https://www.proquest.com/openview/0701fc2d3d8ffd1e46a506c6fe523bfd/1>
8. Bibi, K., Fatima, A., Amin, R., & Rowland, D. L. (2022). Understanding serial mediators of problematic pornography use in Pakistani men and women. *International Journal of Environmental Research and Public Health, 19*(21), 14336. <https://doi.org/10.3390/ijerph192114336>
9. Bohannon, K. E. (2021). Christian women’s pornography usage: The role of perceived addiction, social anxiety, shame, and grace [Doctoral dissertation, Liberty University]. Liberty University Digital Commons. [https://digitalcommons.liberty.edu/doctoral/2841/](https://digitalcommons.liberty.edu/doctoral/2841/?utm_source=chatgpt.com)
10. Borgogna, N. C., Duncan, J., & McDermott, R. C. (2018). Is scrupulosity behind the relationship between problematic pornography viewing and depression, anxiety, and stress? *Sexual Addiction & Compulsivity, 25*(4), 293–318. https://doi.org/10.1080/10720162.2019.1567410
11. Borgogna, N. C., & McDermott, R. C. (2018). The role of gender, experiential avoidance, and scrupulosity in problematic pornography viewing: A moderated-mediation model. *Sexual Addiction & Compulsivity, 25*(4), 319–344. https://doi.org/10.1080/10720162.2018.1503123
12. Borgogna, N. C., & Aita, S. L. (2019). Problematic pornography viewing from a Big Five personality perspective. *Sexual Addiction & Compulsivity, 26*(3–4), 293–314. <https://doi.org/10.1080/10720162.2019.1670302>
13. Borgogna, N. C., McDermott, R. C., Berry, A. T., & Browning, B. R. (2020). Masculinity and problematic pornography viewing: The moderating role of self-esteem. *Psychology of Men & Masculinities, 21*(1), 81–90. <https://doi.org/10.1037/men0000214>
14. Borgogna, N. C., Isacco, A., & McDermott, R. C. (2020). A closer examination of the relationship between religiosity and problematic pornography viewing in heterosexual men. *Sexual Addiction & Compulsivity*, *27*(1-2), 23-44. https://doi.org/10.1080/10720162.2020.1751361
15. Borgogna, N. C., Johnson, D. A., Shegedin, M., &Brasil, K. M. (2024). Taking another look at the pornography problems due to moral incongruence model: Does gender play a role? *Archives of Sexual Behavior, 53*(2), 703–714. https://doi.org/10.1007/s10508-023-02743-y
16. Bőthe, B., Tóth-Király, I., Potenza, M. N., Griffiths, M. D., Orosz, G., & Demetrovics, Z. (2019). Revisiting the role of impulsivity and compulsivity in problematic sexual behaviors. *The Journal of Sex Research, 56*(2), 166–179. https://doi.org/10.1080/00224499.2018.1480744
17. Bőthe, B., Vaillancourt-Morel, M. P., Dion, J., Paquette, M. M., Massé-Pfister, M., Tóth-Király, I., & Bergeron, S. (2022). A longitudinal study of adolescents' pornography use frequency, motivations, and problematic use before and during the COVID-19 pandemic. *Archives of Sexual Behavior, 51*(1), 139–156. https://doi.org/10.1007/s10508-021-02282-4
18. Bradley, D. F., Grubbs, J. B., Uzdavines, A., Exline, J. J., & Pargament, K. I. (2016). Perceived addiction to Internet pornography among religious believers and nonbelievers. *Sexual Addiction & Compulsivity, 23*(2–3), 225–243. https://doi.org/10.1080/10720162.2016.1162237
19. Buczó, D., Koós, M., Nagy, L., Demetrovics, Z., &Bőthe, B. (2025). Further investigation on sexual abuse experiences: Revisiting the psychometric properties of the sexual abuse history questionnaire and the content of its open-ended item. *Sexuality Research and Social Policy, 22*(1), 211–225. https://doi.org/10.1007/s13178-023-00919-8
20. Butler, M. H., Pereyra, S. A., Draper, T. W., Leonhardt, N. D., & Skinner, K. B. (2018). Pornography use and loneliness: A bidirectional recursive model and pilot investigation. *Journal of Sex & Marital Therapy, 44*(2), 127–137. https://doi.org/10.1080/0092623x.2017.1321601
21. Cacioppo, M., Gori, A., Schimmenti, A., Baiocco, R., Laghi, F., & Caretti, V. (2018). Development of a new screening tool for cyber pornography: Psychometric properties of the Cyber Pornography Addiction Test (CYPAT). *Clinical Neuropsychiatry, 15*(1), 60–65. [https://www.clinicalneuropsychiatry.org/download/development-of-a-new-screening-tool-for-cyber-pornography-psychometric-properties-of-the-cyber-pornography-addiction-test-cypa](https://www.clinicalneuropsychiatry.org/download/development-of-a-new-screening-tool-for-cyber-pornography-psychometric-properties-of-the-cyber-pornography-addiction-test-cypat/?utm_source=chatgpt.com)t
22. Calvert, M., Linden, M., Kyser, K., Zeinert, K., & Foust, M. S. (2021). Personality correlates of gaming and pornography use. Psi Beta Journal of Research, 1(1), 34–40. https://doi.org/10.54581/JPWV2620
23. Cardoso, J., Ramos, C., Brito, J., & Almeida, T. C. (2022). Predictors of pornography use: Difficulties in emotion regulation and loneliness. *The Journal of Sexual Medicine, 19*(4), 620–628. <https://doi.org/10.1016/j.jsxm.2022.01.005>
24. Cardoso, J., Ramos, C., Brito, J., & Almeida, T. C. (2023). Difficulties in emotion regulation and problematic pornography use: The mediating role of loneliness. *International Journal of Sexual Health, 35*(3), 481–493. <https://pubmed.ncbi.nlm.nih.gov/38601731/>
25. Chew, P. K., & Poh, E. Y. (2026). Negative emotional states and technological addictions: The buffering and paradoxical role of perceived social support. *Australasian psychiatry : bulletin of Royal Australian and New Zealand College of Psychiatrists*, *34*(1), 31–39. <https://doi.org/10.1177/10398562251358134>
26. DarvishMolla, M., Shirazi, M., &Nikmanesh, Z. (2018). The role of difficulties in emotion regulation and thought control strategies on pornography use. Practice in Clinical Psychology, 6(2), 119–128. https://doi.org/10.29252/nirp.jpcp.6.2.119.
27. Daspe, M.-È., Vaillancourt-Morel, M.-P., Lussier, Y., Sabourin, S., & Ferron, A. (2018). When pornography use feels out of control: The moderation effect of relationship and sexual satisfaction. Journal of Sex & Marital Therapy, 44(4), 343–353. <https://doi.org/10.1080/0092623x.2017.1405301>
28. Drewek. (2020). Correlates and cognitive mechanisms associated with internet pornography use [Master’s thesis, University of Adelaide]. University of Adelaide Digital Library. [https://digital.library.adelaide.edu.au/server/api/core/bitstreams/7ecd5139-7655-4861-8183-b85e5dac38e7/content](https://digital.library.adelaide.edu.au/server/api/core/bitstreams/7ecd5139-7655-4861-8183-b85e5dac38e7/content?utm_source=chatgpt.com)
    **Note:** Author name appears in repository file name; initials not available in the accessible document.
29. Efrati, Y., & Amichai-Hamburger, Y. (2019). The use of online pornography as compensation for loneliness and lack of social ties among Israeli adolescents. Psychological Reports, 122(5), 1865–1882. https://doi.org/10.1177/0033294118797580
30. Egan, V., & Parmar, R. (2013). Dirty habits? Online pornography use, personality, obsessionality, and compulsivity. Journal of Sex & Marital Therapy, 39(5), 394–409. https://doi.org/10.1080/0092623x.2012.710182
31. Etsitty, T. (2022). The influence of maladaptive metacognitions in education: Rethinking proneness toward addiction [Doctoral dissertation, Wichita State University]. SOAR: Shocker Open Access Repository. [https://soar.wichita.edu/entities/publication/57f9512e-56d4-45f9-bde7-e60f13c8956](https://soar.wichita.edu/entities/publication/57f9512e-56d4-45f9-bde7-e60f13c89562?utm_source=chatgpt.com)2
32. Ferron, A., Lussier, Y., Sabourin, S., & Brassard, A. (2016). The role of internet pornography use and cyber infidelity in the associations between personality, attachment, and couple and sexual satisfaction. Social Networking, 6(1), 1–18. <https://doi.org/10.4236/sn.2016.61001>
33. Floyd, C. G., Volk, F., Flory, D., Harden, K., Peters, C. E., & Taylor, A. (2022). Sexual shame as a unique distress outcome of morally incongruent pornography use: Modifications and methodological considerations. Archives of Sexual Behavior, 51(2), 1293–1311. https://doi.org/10.1007/s10508-021-02104-7
34. Galán, M., Pineda, D., Rico-Bordera, P., Martínez-Martínez, A., & Piqueras, J. A. (2024). The influence of dark personality and pornography on sexual aggression beliefs. Frontiers in Psychology, 15, 1471438. https://doi.org/10.3389/fpsyg.2024.1471438
35. Gerymski, R. (2017). Are personality traits good predictors of the type of watched pornography? Analysis of the relationship between personality traits and the preference of chosen pornographic categories. PrzeglądSeksuologiczny, 15(3), 10–16. https://repo.uni.opole.pl/info/article/UO85daef29b1d7477b9324076def1cf2c1/
36. Gewirtz-Meydan, A., Mitchell, K. J., Spivak-Lavi, Z., & Kraus, S. W. (2021). Attachment insecurities and body image self-consciousness among women: The mediating role of pornography use. Computers in Human Behavior, 124, 106916. <https://doi.org/10.1016/j.chb.2021.106916>
37. Grubbs, J. B., Wilt, J. A., Exline, J. J., Pargament, K. I., & Kraus, S. W. (2018). Moral disapproval and perceived addiction to internet pornography: a longitudinal examination. *Addiction (Abingdon, England)*, *113*(3), 496–506. https://doi.org/10.1111/add.14007
38. Grubbs, J. B., Wilt, J. A., Exline, J. J., & Pargament, K. I. (2018). Predicting pornography use over time: Does self-reported “addiction” matter? Addictive Behaviors, 82, 57–64. <https://doi.org/10.1016/j.addbeh.2018.02.028>
39. Grubbs, J. B., Stauner, N., Exline, J. J., Pargament, K. I., & Lindberg, M. J. (2015). Perceived addiction to Internet pornography and psychological distress: Examining relationships concurrently and over time. *Psychology of addictive behaviors : journal of the Society of Psychologists in Addictive Behaviors*, *29*(4), 1056–1067. <https://doi.org/10.1037/adb0000114>
40. Grubbs, J. B., Kraus, S. W., & Perry, S. L. (2019). Self-reported addiction to pornography in a nationally representative sample: The roles of use habits, religiousness, and moral incongruence. *Journal of behavioral addictions*, *8*(1), 88–93. <https://doi.org/10.1556/2006.7.2018.134>
41. Grubbs, J. B., Grant, J. T., & Engelman, J. (2018). Self-identification as a pornography addict: Examining the roles of pornography use, religiousness, and moral incongruence. *Sexual Addiction & Compulsivity*, *25*(4), 269-292. https://doi.org/10.1080/10720162.2019.1565848
42. Guidry, R., Floyd, C. G., Volk, F., & Moen, C. E. (2020). The exacerbating impact of moral disapproval on the relationship between pornography use and depression, anxiety, and relationship satisfaction. Journal of Sex & Marital Therapy, 46(2), 103–121. https://doi.org/10.1080/0092623x.2019.1654579
43. Hardani, R., Hastuti, D., &Yuliati, L. N. (2018). Pornography behavior of junior high school students. Journal of Child Development Studies, 3(1), 15–27. <https://doi.org/10.29244/jcds.3.1.15-27>
44. Hassanzadeh, M., Omidi, A., & Zanjani, Z. (2025). Prediction of problematic pornography use based on personality traits with mediating role of impulsivity: A structural equation modeling modeling approach abstract. Iranian Journal of Psychiatry and Behavioral Sciences, 19(3), e159063. [https://doi.org/10.5812/ijpbs-15906](https://doi.org/10.5812/ijpbs-159063)3
45. Harper, C., & Hodgins, D. C. (2016). Examining correlates of problematic internet pornography use among university students. Journal of Behavioral Addictions, 5(2), 179–191. <https://doi.org/10.1556/2006.5.2016.022>
46. Hew, Y. C., & Chew, P. K. (2026). Distress tolerance as a risk factor for specific Internet-use disorders. *Journal of Technology in Behavioral Science*, *11*(1), 71-82. <https://doi.org/10.1007/s41347-025-00489-z>
47. Hughes, A., Brewer, G., & Khan, R. (2020). Sexual Coercion by Women: The Influence of Pornography and Narcissistic and Histrionic Personality Disorder Traits. *Archives of sexual behavior*, *49*(3), 885–894. https://doi.org/10.1007/s10508-019-01538-4
48. Huțul, T. D., Karner-Huțuleac, A., Mariș, A. M., Filote, I. M., Chiricheș, F. B., &Mavrichi, M. I. F. (2025). The role of positive and negative metacognitions about sex in the relationship between psychological distress and problematic consumption of pornography: A mediation model. International Journal of Sexual Health, 37(1), 30–45. https://doi.org/10.1080/19317611.2024.2410943
49. Huțul, T. D., Karner-Huțuleac, A., Huțul, A., & Popescu, A. (2024). Examination of gender differences in depression, relationship satisfaction, and problematic pornography use among Romanian gay men and lesbian women. International Journal of Sexual Health, 36(2), 205–220. https://doi.org/10.1080/19317611.2024.2329973
50. Huțul, T. D., Huțul, A., & Karner-Huțuleac, A. (2025). Attachment styles as mediators between self-perceived addiction to pornography and psychological distress: Findings and implications from a Romanian cross-sectional study. Logos Universality Mentality Education Novelty: Social Sciences, 14(1), 52–71. https://doi.org/10.18662/lumenss/14.1/115
51. Jain, S., & Pandey, N. (2020). Exploring the connection between problematic pornography, personality, and emotional adjustment. International Journal of Current Research and Review, 12(20), 173–179. https://doi.org/10.31782/IJCRR.2020.122023
52. Jiang, X., Lu, Y., Hong, Y., Zhang, Y., & Chen, L. (2022). A network comparison of motives behind online sexual activities and problematic pornography use during the COVID-19 outbreak and the post-pandemic period. International Journal of Environmental Research and Public Health, 19(10), 5870. <https://doi.org/10.3390/ijerph19105870>
53. Khaleghian, A., Sajjadian, I., Fatehizade, M., & Manshaei, G. R. (2020). The mediating role of impulsivity and experiential avoidance in the relationship between emotion regulation and pornography viewing in married men. http://jpcp.uswr.ac.ir/article-1-646-fa.html
54. Khan, A., Latif, M., Laghari, I. A., Alvi, W. I., & Riaz, H. (2024). The impact of pornography on psychological distress and loneliness among university students of Karachi, Pakistan: An explorative study. Journal of Social Sciences Research & Policy, 2(3), 190–198. https://jssrp.org.pk/index.php/jssrp/article/view/98
55. Leonhardt, N. D., Willoughby, B. J., & Young-Petersen, B. (2018). Damaged goods: Perception of pornography addiction as a mediator between religiosity and relationship anxiety surrounding pornography use. The Journal of Sex Research, 55(3), 357–368. https://doi.org/10.1080/00224499.2017.1295013
56. Levin, M. E., Lillis, J., & Hayes, S. C. (2012). When is online pornography viewing problematic among college males? Examining the moderating role of experiential avoidance. Sexual Addiction & Compulsivity, 19(3), 168–180. https://doi.org/10.1080/10720162.2012.657150
57. Lewczuk, K., Wizła, M., & Gola, M. (2023). The relation of sexual attitudes to hypersexuality and problematic pornography use. Archives of Sexual Behavior, 52(1), 411–430. https://doi.org/10.1007/s10508-022-02358-9
58. Li, L., Wang, X., Tang, S., & Wang, J. (2023). Family functioning and problematic internet pornography use among adolescents: A moderated mediation model. Frontiers in Public Health, 11, 1199835. https://doi.org/10.3389/fpubh.2023.1199835
59. Malaeb, D., Hallit, S., & Obeid, S. (2023). Pornography use among Lebanese adults: Association with loneliness and fear of commitment. Healthcare, 11(6), 795. https://doi.org/10.3390/healthcare11060795
60. Maitland, D. W., & Neilson, E. C. (2023). Associations between pornography consumption patterns, pornography consumption motives, and social wellbeing among US college students: A latent profile analysis with a primarily female sample. Journal of Sex & Marital Therapy, 49(7), 739–754. https://doi.org/10.1080/0092623X.2023.2193182
61. Marino, C., Melodia, F., Pivetta, E., Mansueto, G., Palmieri, S., Caselli, G., ... & Spada, M. M. (2023). Desire thinking and craving as predictors of problematic internet pornography use in women and men. Addictive Behaviors, 136, 107469. https://doi.org/10.1016/j.addbeh.2022.107469
62. Maas, M. K., Vasilenko, S. A., & Willoughby, B. J. (2018). A dyadic approach to pornography use and relationship satisfaction among heterosexual couples: The role of pornography acceptance and anxious attachment. The Journal of Sex Research, 55(6), 772–782. <https://doi.org/10.1080/00224499.2018.1440281>
63. Miller, K. D. (2020). The effect of solo-masturbation and moral disapproval on the relationship between Internet pornography use, religious struggles, sexual shame, and depression: A moderated mediation model [Doctoral dissertation, Liberty University]. Liberty University Digital Commons. [https://digitalcommons.liberty.edu/doctoral/276](https://digitalcommons.liberty.edu/doctoral/2765/)5
64. Müller, S. M., & Antons, S. (2023). Decision making and executive functions in problematic pornography use. Frontiers in Psychiatry, 14, 1191297. https://doi.org/10.3389/fpsyt.2023.1191297
65. Muris, P., Otgaar, H., Meesters, C., Papasileka, E., & Pineda, D. (2020). The dark triad and honesty-humility: A preliminary study on the relations to pornography use. Dignity: A Journal of Analysis of Exploitation and Violence, 5(1), Article 3. https://doi.org/10.23860/dignity.2020.05.01.03
66. Musetti, A., Gori, A., Alessandra, A., Topino, E., Terrone, G., Plazzi, G., ... & Franceschini, C. (2022). The interplay between problematic online pornography use, psychological stress, emotion dysregulation, and insomnia symptoms during the COVID-19 pandemic: A mediation analysis. Nature and Science of Sleep, 14, 83–92. https://doi.org/10.2147/nss.s348242
67. Naeem, S., & Zulfiqar, U. (2025). Stress, hypersexuality, and problematic pornography consumption among young drug addicts of Pakistan. Journal of Psychology, Health and Social Challenges, 3(2), 49–58. https://doi.org/10.63075/hwn5e519
68. Niazof, D., Weizman, A., & Weinstein, A. (2019). The contribution of ADHD and attachment difficulties to online pornography use among students. Comprehensive Psychiatry, 93, 56–60. <https://doi.org/10.1016/j.comppsych.2019.07.002>
69. Ostrander, M. J. (2021). Social anxiety, pornography use, and loneliness: A mediation analysis [Doctoral dissertation, Liberty University]. Liberty University Digital Commons. [https://digitalcommons.liberty.edu/doctoral/294](https://digitalcommons.liberty.edu/doctoral/2940/?utm_source=chatgpt.com)0
70. Pepping, C. A., Cronin, T. J., Lyons, A., & Caldwell, J. G. (2018). The effects of mindfulness on sexual outcomes: The role of emotion regulation. Archives of Sexual Behavior, 47(6), 1601–1612. <https://doi.org/10.1007/s10508-017-1127-x>
71. Pereyra, S. A. (2016). Pornography use and loneliness: Assessing correlations using three associative models [Master’s thesis, Brigham Young University]. BYU ScholarsArchive. [https://scholarsarchive.byu.edu/etd/641](https://scholarsarchive.byu.edu/etd/6417/?utm_source=chatgpt.com)7
72. Perez del Valle, J., & Hand, C. J. (2022). The role of scrupulosity, experiential avoidance, and the Dark Tetrad in problematic pornography use. Sexual Health & Compulsivity, 29(1–2), 68–95. <https://doi.org/10.1080/26929953.2022.2101168>
73. Rimti, F. H., Ripon, R. K., Al Zubayer, A., Rimu, F. H., Rahman, Q. M., Nailah, M. T., ... & Hawlader, M. D. H. (2025). Examining the relationship between problematic pornography use and mental health status among adolescents of Bangladesh [Preprint]. Research Square. [https://doi.org/10.21203/rs.3.rs-3870491/v](https://doi.org/10.21203/rs.3.rs-3870491/v2)2
74. Russo, A., Santoro, G., & Schimmenti, A. (2022). Interpersonal guilt and problematic online behaviors: The mediating role of emotion dysregulation. Clinical Neuropsychiatry, 19(4), 236–245. https://doi.org/10.36131/cnfioritieditore20220406
75. Sassover, E., Abrahamovitch, Z., Amsel, Y., Halle, D., Mishan, Y., Efrati, Y., & Weinstein, A. (2023). A study on the relationship between shame, guilt, self-criticism, and compulsive sexual behaviour disorder. Current Psychology, 42(10), 8347–8355. https://doi.org/10.1007/s12144-021-02188-3
76. Sfeir, E., Malaeb, D., Hallit, S., & Obeid, S. (2022). Personality traits and online pornography use among Lebanese adults. The Primary Care Companion for CNS Disorders, 24(6), 21m03439. <https://doi.org/10.4088/pcc.22m03131>
77. Shelton, D. L. (2019). Moral incongruence and perceived addiction: A contemporary dilemma with pornography use [Doctoral dissertation, Liberty University]. Liberty University Digital Commons. [https://digitalcommons.liberty.edu/doctoral/207](https://digitalcommons.liberty.edu/doctoral/2078/?utm_source=chatgpt.com)8
78. Short, M. B., Kasper, T. E., &Wetterneck, C. T. (2015). The relationship between religiosity and internet pornography use. Journal of Religion and Health, 54(2), 571–583. https://doi.org/10.1007/s10943-014-9849-8
79. Siddik, M. A. B., Pervin, I., Syfullah, M. K., Ali, A., Mahmud, A., Hasan, M., ... & Rahman, M. M. (2024). Post-COVID-19 internet addiction, depression, and pornography addiction among adolescents: Findings from a nationwide study in Bangladesh. Health Science Reports, 7(7), e2272. https://doi.org/10.1002/hsr2.2272
80. Su, Y., Zheng, L., & Zheng, Y. (2024). Pornography use and mental health problems in the Chinese population: Examining the pornography problems due to moral incongruence model. The Journal of Sex Research, 61(8), 1210–1221. https://doi.org/10.1080/00224499.2023.2201255
81. Tan, S. A., Goh, Y. S., Mohd Zaharim, N., Gan, S. W., Yap, C. C., Nainee, S., & Lee, L. K. (2022). Problematic internet pornography use and psychological distress among emerging adults in Malaysia: Gender as a moderator. International Journal of Environmental Research and Public Health, 19(6), 3682. https://doi.org/10.3390/ijerph19063682
82. Rømer Thomsen, K., Callesen, M. B., Hesse, M., Kvamme, T. L., Pedersen, M. M., Pedersen, M. U., & Voon, V. (2018). Impulsivity traits and addiction-related behaviors in youth. Journal of Behavioral Addictions, 7(2), 317–330. https://doi.org/10.1556/2006.7.2018.22
83. Tian, Y., Zhang, S., Wu, R., Wang, P., Gao, F., & Chen, Y. (2018). Association between specific internet activities and life satisfaction: The mediating effects of loneliness and depression. Frontiers in Psychology, 9, 1181. <https://doi.org/10.3389/fpsyg.2018.01181>
84. Twine, S. L. (2015). Attachment and pornography use: The influence of romantic attachment styles, intimacy, and pornography use on marital satisfaction [Doctoral dissertation, Liberty University]. Liberty University Digital Commons. [https://digitalcommons.liberty.edu/doctoral/109](https://digitalcommons.liberty.edu/doctoral/1093/?utm_source=chatgpt.com)3
85. Udoh, E. A., Azubike, A. O., & Ajibola, A. R. (2023). Demographics, sexual satisfaction, and self-control as predictors of pornography consumption among married persons in Uyo Metropolis. International Journal of Psychiatry, 8(5), 175–179. https://doi.org/10.31579/2637-8892/231
86. Ugese, I. J., Chinonso, U., Gandi, J. C., Shaapera, P. T., Mgbeanuli, C. C., Ubani, C. A., ... & Balarabe, M. B. (2024). Pornography addiction in emerging adults: The role of social isolation, self-control, and stress coping. British Journal of Psychology Research, 12(1), 26–36. https://doi.org/10.37745/bjpr.2013/vol12n12636
87. Vescan, M., Flack, M., & Caudwell, K. M. (2024). Loneliness and problematic pornography use: What is the role of emotion regulation and interaction with content creators? Addictive Behaviors Reports, 19, 100550. <https://doi.org/10.1016/j.abrep.2024.100550>
88. Volk, F., Thomas, J., Sosin, L., Jacob, V., & Moen, C. (2016). Religiosity, developmental context, and sexual shame in pornography users: A serial mediation model. Sexual Addiction & Compulsivity, 23(2–3), 244–259. https://doi.org/10.1080/10720162.2016.1151391
89. Volk, F., Floyd, C. G., Bohannon, K. E., Cole, S. M., McNichol, K. M., Schott, E. A., & Williams, Z. D. (2019). The moderating role of the tendency to blame others in the development of perceived addiction, shame, and depression in pornography users. Sexual Addiction & Compulsivity, 26(3–4), 239–261. https://doi.org/10.1080/10720162.2019.1670301
90. Wilt, J. A., Cooper, E. B., Grubbs, J. B., Exline, J. J., & Pargament, K. I. (2016). Associations of perceived addiction to internet pornography with religious/spiritual and psychological functioning. Sexual Addiction & Compulsivity, 23(2–3), 260–278. https://doi.org/10.1080/10720162.2016.1140604
91. Wizła, M., &Lewczuk, K. (2024). The associations between attachment insecurity and compulsive sexual behavior disorder or problematic pornography use: The mediating role of emotion regulation difficulties. Archives of Sexual Behavior, 53(9), 3419–3436. https://doi.org/10.1007/s10508-024-02904-7
92. Yundianto, D., & Abulkhair, A. G. (2023). When homework makes you compulsive: Determining the role of academic stress to cyberpornography use with gender as comparing variable. MOJPC: Malaysia Online Journal of Psychology & Counselling, 10(1), 24–33. [https://mjcs.um.edu.my/index.php/MOJC/article/view/4510](https://mjcs.um.edu.my/index.php/MOJC/article/view/45107)7
93. Zhang, Y., Chen, L., Jiang, X., &Bőthe, B. (2022). Investigating the associations of ADHD symptoms, impulsivity, physical exercise, and problematic pornography use in a Chinese sample. International Journal of Environmental Research and Public Health, 19(22), 15221. <https://doi.org/10.3390/ijerph192215221>
94. Asadi, F. (2021). *Barrasi-ye rabete-ye rezayat-e jensi va gerayesh be pornografi ba rezayatmandi-ye zanashuyi-ye karkonan-e eqmari* [Examining the relationship of sexual satisfaction and tendency toward pornography with marital satisfaction among rotational employees] [Master’s thesis, Alzahra University]. Faculty of Education and Psychology. [In Persian; title translated from Persian ]
95. Beyramvand, Y. (2019). Pishbini-ye estefadeh az harzeh-negari-ye interneti bar asas-e vizhegi-haye shakhsiati va keyfiyat-e ravabet-e zanashuyi dar mota’ahhelin-e shahr-e Esfahan [Predicting internet pornography use based on personality traits and marital relationship quality among married individuals in Isfahan] [Master’s thesis, Yazd University]. Faculty of Education and Psychology, Campus of Humanities and Social Sciences. [In Persian; title translated from Persian
96. Pourjafari, M. (2021). *Naghsh-e karkard-e khanevadeh, ehsas-e tanhaei va maharat-e ertebati dar pishbini-ye etiad be harzeh-negari dar beyn-e daneshjuyan* [The role of family functioning, loneliness, and communication skills in predicting pornography addiction among students]. *Journal of Psychology New Ideas, 8*(12), 1–11. [In Persian; title translated from Persian ]
97. Tanha, Z., & Babaei Motlagh, F. (2022). Rabete-ye enzavaye fardi va etiad be internet ba gerayesh be raftarhaye pornografi dar dowran-e corona [The relationship of individual isolation and internet addiction with tendency toward pornographic behaviors during the COVID-19 pandemic]. Women Interdisciplinary Researches, 4(3), 75–86. [In Persian; title translated from Persian ]

**Table 5.** Categorical Moderator Results

| **Variables** | **Moderator** | **k** | **N** | **r** | **r_ci.lb** | **r_ci.ub** | **Z** | **Q** | **I2** |
| --- | --- | --- | --- | --- | --- | --- | --- | --- | --- |
| Anxiety |  |  |  |  |  |  |  |  |  |
|  | COVID19 | (Q = 1.76, df = 1, p = 0.18) | | | | | | | |
|  | pre | 6 | 4634 | 0.10 | 0.03 | 0.17 | 2.85* | 38.02 | 75.33 |
|  | during | 7 | 2599 | 0.17 | 0.10 | 0.23 | 1.33 | 38.02 | 75.33 |
|  | **PPU instrument**  **type** | (Q = 0.00, df = 1, p = 0.98) | | | | | | | |
|  | Dysregulation-oriented | 6 | 2324 | 0.19 | 0.10 | 0.28 | 3.99 | 46.16 | 83.76 |
|  | Distress-oriented | 5 | 3661 | 0.19 | 0.10 | 0.28 | -0.02 | 46.16 | 83.76 |
| Coping |  |  |  |  |  |  |  |  |  |
|  | COVID19 | (Q = 1.69, df = 1, p = 0.19) | | | | | | | |
|  | post | 4 | 597 | 0.16 | 0.02 | 0.30 | 2.30* | 228.01 | 94.52 |
|  | during | 7 | 5488 | 0.00 | -0.20 | 0.20 | -1.30 | 228.01 | 94.52 |
| Depression |  |  |  |  |  |  |  |  |  |
|  | COVID19 | (Q = 2.56, df = 2, p = 0.28) | | | | | | | |
|  | pre | 6 | 9299 | 0.18 | -0.06 | 0.39 | 1.49 | 705.24 | 98.22 |
|  | during | 7 | 2117 | 0.39 | 0.19 | 0.57 | 1.43 | 705.24 | 98.22 |
|  | post | 4 | 9505 | 0.41 | 0.13 | 0.62 | 1.29 | 705.24 | 98.22 |
|  | **PPU instrument**  **type** | (Q = 0.68, df = 1, p = 0.41) | | | | | | | |
|  | Dysregulation-oriented | 8 | 15907 | 0.28 | 0.03 | 0.50 | 2.17 | 2578.51 | 99.06 |
|  | Distress-oriented | 6 | 3803 | 0.41 | 0.21 | 0.58 | 0.83 | 2578.51 | 99.06 |
| Family environment |  |  |  |  |  |  |  |  |  |
|  | **PPU instrument**  **type** | (Q = 0.27, df = 1, p = 0.61) | | | | | | | |
|  | Dysregulation-oriented | 4 | 3203 | 0.039 | -0.153 | 0.227 | 0.393 | 187.758 | 95.593 |
|  | Distress-oriented | 6 | 1206 | -0.040 | -0.263 | 0.186 | -0.517 | 187.758 | 95.593 |
| Impulsivity |  |  |  |  |  |  |  |  |  |
|  | Country | (Q = 0.09, df = 1, p = 0.77) | | | | | | | |
|  | China | 5 | 3500 | 0.20 | 0.01 | 0.37 | -0.30 | 260.60 | 97.16 |
|  | Germany | 6 | 4800 | 0.24 | 0.04 | 0.41 | 2.38* | 260.60 | 97.16 |
|  | COVID19 | (Q = 0.15, df = 1, p = 0.70) | | | | | | | |
|  | post | 6 | 1212 | 0.25 | 0.12 | 0.37 | 3.68*** | 370.54 | 98.14 |
|  | during | 12 | 23072 | 0.20 | 0.01 | 0.38 | -0.39 | 370.54 | 98.14 |
| Loneliness |  |  |  |  |  |  |  |  |  |
|  | COVID19 | (Q = 1.18, df = 2, p = 0.56) | | | | | | | |
|  | pre | 5 | 12744 | 0.17 | 0.02 | 0.30 | 2.25* | 207.46 | 95.59 |
|  | during | 6 | 2748 | 0.22 | 0.09 | 0.35 | 0.55 | 207.46 | 95.59 |
|  | post | 10 | 3618 | 0.26 | 0.16 | 0.36 | 1.08 | 207.46 | 95.59 |
|  | PPU**PPU instrument**  **type** | (Q = 4.69, df = 1, p = 0.03) | | | | | | | |
|  | Dysregulation-oriented | 12 | 8825 | 0.12 | -0.03 | 0.26 | 1.57 | 146.11 | 93.27 |
|  | Distress-oriented | 4 | 6077 | 0.30 | 0.22 | 0.38 | 2.17 | 146.11 | 93.27 |
| Moral disapproval |  |  |  |  |  |  |  |  |  |
|  | COVID19 | (Q = 0.15, df = 1, p = 0.70) | | | | | | | |
|  | pre | 4 | 7324 | 0.23 | -0.02 | 0.46 | 1.77 | 2231.18 | 99.33 |
|  | during | 11 | 6704 | 0.32 | -0.10 | 0.64 | 0.38 | 2231.18 | 99.33 |
| Neuroticism |  |  |  |  |  |  |  |  |  |
|  | **PPU instrument**  **type** | (Q = 14.22, df = 1, p = 0.00) | | | | | | | |
|  | Dysregulation-oriented | 4 | 1870 | 0.27 | 0.23 | 0.32 | 11.79 | 14.64 | 34.96 |
|  | Distress-oriented | 6 | 3267 | 0.13 | 0.08 | 0.19 | -3.77 | 14.64 | 34.96 |
| Religiousness | |  |  |  |  |  |  |  |  |
|  | COVID19 | (Q = 0.59, df = 2, p = 0.74) | | | | | | | |
|  | pre | 7 | 11316 | 0.06 | -0.17 | 0.28 | 0.48 | 1764.80 | 98.93 |
|  | during | 7 | 5163 | 0.18 | -0.05 | 0.40 | 0.77 | 1764.80 | 98.93 |
|  | post | 4 | 2515 | 0.12 | -0.19 | 0.40 | 0.30 | 1764.80 | 98.93 |
|  | **PPU instrument**  **type** | (Q = 4.45, df = 1, p = 0.04) | | | | | | | |
|  | Dysregulation-oriented | 4 | 2515 | 0.44 | 0.25 | 0.60 | 4.18 | 262.10 | 97.90 |
|  | Distress-oriented | 5 | 6327 | 0.12 | -0.13 | 0.35 | -2.11 | 262.10 | 97.90 |
| Stress |  |  |  |  |  |  |  |  |  |
|  | COVID19 | (Q = 4.88, df = 1, p = 0.03) | | | | | | | |
|  | pre | 4 | 3398 | 0.28 | 0.18 | 0.37 | 5.33*** | 31.81 | 83.09 |
|  | post | 6 | 1590 | 0.12 | 0.01 | 0.22 | -2.21* | 31.81 | 83.09 |
|  | PPU instrument  type | (Q = 0.61, df = 1, p = 0.44) | | | | | | | |
|  | Dysregulation-oriented | 7 | 3370 | 0.13 | -0.05 | 0.29 | 1.42 | 101.41 | 93.11 |
|  | Distress-oriented | 4 | 2695 | 0.21 | 0.08 | 0.33 | 0.78 | 101.41 | 93.11 |
|  |  |  |  |  |  |  |  |  |  |

Note. k = number of studies; N = number of participants; r = mean weighted effect size; CI= confidence interval; z=z value of the significance test; Q = ratio of variation to within-study error; *I^2^* = proportion of total observed variation attributable to between-study effects. **p*<.05. ***p*<.01. ****p*<.001
